# Inhibitors of *Trypanosoma cruzi* Sir2 related protein 1 as potential drugs against Chagas disease

**DOI:** 10.1371/journal.pntd.0006180

**Published:** 2018-01-22

**Authors:** Luís Gaspar, Ross P. Coron, Paul KongThoo Lin, David M. Costa, Begoña Perez-Cabezas, Joana Tavares, Meritxell Roura-Ferrer, Isbaal Ramos, Céline Ronin, Louise L. Major, Fabrice Ciesielski, Iain K. Pemberton, Jane MacDougall, Paola Ciapetti, Terry K. Smith, Anabela Cordeiro-da-Silva

**Affiliations:** 1 i3S–Instituto de Investigação e Inovação em Saúde, Universidade do Porto, Porto, Portugal; 2 IBMC-Instituto de Biologia Molecular e Celular, Parasite Disease Group, Porto, Portugal; 3 Biomedical Sciences Research Complex, The University, St. Andrews, Fife. Scotland, United Kingdom; 4 Robert Gordon University, School of Pharmacy and Life Sciences, Aberdeen, Scotland, United Kingdom; 5 Innoprot SL, Derio, Spain; 6 NovAliX, Illkirch Cedex, France; 7 Photeomix Protein Discovery, IP Research Consulting, Noisy Le Grand, France; 8 Departmento de Ciências Biológicas, Faculdade de Farmácia, Universidade do Porto, Porto, Portugal; University of Texas at El Paso, UNITED STATES

## Abstract

Chagas disease remains one of the most neglected diseases in the world despite being the most important parasitic disease in Latin America. The characteristic chronic manifestation of chagasic cardiomyopathy is the region’s leading cause of heart-related illness, causing significant mortality and morbidity. Due to the limited available therapeutic options, new drugs are urgently needed to control the disease. Sirtuins, also called Silent information regulator 2 (Sir2) proteins have long been suggested as interesting targets to treat different diseases, including parasitic infections. Recent studies on *Trypanosoma cruzi* sirtuins have hinted at the possibility to exploit these enzymes as a possible drug targets. In the present work, the *T*. *cruzi* Sir2 related protein 1 (TcSir2rp1) is genetically validated as a drug target and biochemically characterized for its NAD^+^-dependent deacetylase activity and its inhibition by the classic sirtuin inhibitor nicotinamide, as well as by bisnaphthalimidopropyl (BNIP) derivatives, a class of parasite sirtuin inhibitors. BNIPs ability to inhibit TcSir2rp1, and anti-parasitic activity against *T*. *cruzi* amastigotes *in vitro* were investigated. The compound BNIP Spermidine (BNIPSpd) (**9**), was found to be the most potent inhibitor of TcSir2rp1. Moreover, this compound showed altered trypanocidal activity against TcSir2rp1 overexpressing epimastigotes and anti-parasitic activity similar to the reference drug benznidazole against the medically important amastigotes, while having the highest selectivity index amongst the compounds tested. Unfortunately, BNIPSpd failed to treat a mouse model of Chagas disease, possibly due to its pharmacokinetic profile. Medicinal chemistry modifications of the compound, as well as alternative formulations may improve activity and pharmacokinetics in the future. Additionally, an initial TcSIR2rp1 model in complex with p53 peptide substrate was obtained from low resolution X-ray data (3.5 Å) to gain insight into the potential specificity of the interaction with the BNIP compounds. In conclusion, the search for TcSir2rp1 specific inhibitors may represent a valuable strategy for drug discovery against *T*. *cruzi*.

## Author summary

*Trypanosoma cruzi* is a protozoan parasite belonging to the Kinetoplastida class responsible for Chagas disease, a neglected tropical illness that affects an estimated 6 to 8 million people in Latin America and some Southern regions of the USA, with another 25 million at risk of acquiring the disease and a death toll of 12,000 every year. Commonly transmitted from the feces of the kissing bug, the disease is characterized by a nearly asymptomatic acute phase but a problematic chronic phase in which 20–30% of individuals develop serious cardiac and/or intestinal problems. The therapies currently in use were introduced more than forty years ago, and there are important concerns about adverse effects and lower effectiveness with disease progression. There is, therefore, an urgent need to find better alternatives. In this study, we evaluate the potential of a *Trypanosoma cruzi* sirtuin protein as a novel drug target and its inhibition by novel members of a known class of sirtuin compound inhibitors.

## Introduction

Chagas disease, caused by the protozoan *Trypanosoma cruzi*, remains one of the most prevalent and neglected diseases in the world [1] and is the parasitic disease with the highest socio-economic burden in Latin America [2, 3]. More recently, Chagas disease has also emerged as a significant public health concern imparting a serious burden of disease in some Southern regions of the USA [4]. While the vast majority of the newly acquired cases are asymptomatic, about 20 to 30% of the cases lead to the development, usually decades later, of the characteristic chronic chagasic cardiomyopathy that is the leading cause of non-ischemic heart disease in South America [5, 6].

Although vectorial transmission has been greatly reduced in recent years, mainly due to improved housing and awareness, other transmission mechanisms like congenital or oral transmission, blood transfusion and organ transplantation still contribute to about 40,000 new cases and 12,000 deaths every year [7]. What used to be a local concern, has become a worldwide problem due to human migrations to other parts of the world such as North America, Europe and Japan [8].

With no available vaccine for Chagas disease therapies rely upon two drugs introduced more than 40 years ago: benznidazole and nifurtimox. These drugs are far from ideal because of their many toxic side-effects and the requirement of long periods of administration [4, 9, 10]. Thus, there is an urgent need for new drugs. Very few candidates have populated the drug discovery pipeline for Chagas disease in the past decade, and most have the same mechanism of action [11]. The recent failure in clinical trials of two of these drugs, posaconazole and E1224, has exposed the fragilities of the chemotherapeutic pipeline and reinforced the need for constant drug discovery efforts to find novel alternative targets and therapies [12–14].

Sir2 (silent information regulator 2) or sirtuins are class III histone deacetylases that are evolutionary conserved and present across various kingdoms of life, from bacteria to humans [15, 16]. They catalyse the NAD^+^- dependent deacetylation of acetylated lysine residues in a polypeptide chain, according to the following reaction:

Acetylated-Protein + NAD^+^ → Deacetylated Protein + 2’-O-AADPr + Nicotinamide

Besides the deacetylated lysine, other products are formed in the reaction, like nicotinamide (an endogenous inhibitor) and 2’-O-acetyl-ADP-ribose (2’-O-AADPr), that has been suggested to act as a secondary messenger [17], promoting the association between Sir3/Sir2/Sir4 involved in yeast gene silencing [18]. Some human sirtuins like SIRT4 and SIRT6 and the protozoan Sir2 related protein 1 (Sir2rp1) from *Trypanosoma brucei* and *Leishmania infantum* also display ADP-ribosyltransferase activity [19–22]. However, the biological role of ADP-ribosylation by sirtuins has not been clearly demonstrated and is currently debated to be a non-specific side reaction [23]. In addition, some sirtuins have been characterized to perform demalonylase, desuccinylase and deglutarylase activity [24, 25].

Sirtuins have been attributed many roles in different organisms, including life span regulation, cell cycle progression, gene transcription, apoptosis, DNA repair and metabolism [26–29]. The human genome, as well as other mammals’ genomes, codify 7 distinct sirtuins (SIRT1-7) [30], whose proteins are distributed in different cell compartments: SIRT1, SIRT6 and SIRT7 are located in the nucleus, SIRT2 is cytoplasmic (but shuttles to and from the nucleus), whereas SIRT3, SIRT4 and SIRT5 are found in the mitochondria [31]. Their different localizations are related with their cellular functions, for instance: nuclear SIRT1, 6 and 7 are involved in transcription regulation [32], DNA repair [33, 34] and chromatin remodeling [35, 36], respectively; SIRT2 is a tubulin deacetylase that co-localizes with the cytoskeleton, but is imported to the nucleus, where it participates in cell cycle regulation [37]; and mitochondrial SIRT3, 4 and 5, participate in fatty acid metabolism [38], amino acid metabolism [19] and the urea cycle, respectively [39].

By contrast, parasitic protozoa have fewer sirtuin homologues, but they have also been described as “pro-life” proteins due to their importance for the normal development and functioning of these cells. For instance, in the apicomplexan *Plasmodium falciparum*, Sir2A and B are involved in antigenic variation, an essential process for immune system evasion [40, 41]. Trypanosomatids have two to three homologues (Sir2rp1-3) with some of the members varying in their functions and subcellular localizations. *T*. *brucei* Sir2rp1 is the most characterized of the three enzymes, and is located in the nucleus and seems to be important for the protection against DNA damage [21, 42]. TbSir2rp2 and TbSir2rp3 are localized in the mitochondria and little is known about their function, except that they are not essential for parasite survival [42]. *Leishmania* species also encode for three sirtuin enzymes, but most studies are focused on Sir2rp1, that has a cytoplasmic localization. Sir2rp1 from *L*. *infantum* is required for the normal replication of amastigotes [43]. *T*. *cruzi* genomes sequenced so far, have only revealed two homologues, Sir2rp1 and Sir2rp3, which are localized in the cytoplasm and the mitochondria, respectively [44, 45]. Overexpression of either TcSir2rp1 or TcSir2rp3 showed them to interfere with epimastigote growth, differentiation into metacyclic trypomastigotes, infectivity of host cells and intracellular amastigote replication [44, 45]. It should be noted that some contradictory findings have been reported by both research groups, differences that are likely related with the strategy of overexpression (constitutive [44] versus inducible [45]). A clearer role of the participation of Sir2rp1 and Sir2rp3 in *T*. *cruzi* life cycle and infectivity by gene knockout studies awaits investigation.

Here, we characterize for the first time the enzymatic NAD^+^-dependent deacetylase activity of TcSir2rp1 and show that as well as being hindered by classic inhibitors, bisnaphthalimidopropyl (BNIP) derivatives [46], some showing specific “on target” trypanocidal activity against *T*. *cruzi* epimastigotes are also effective inhibitors. Furthermore, we demonstrate that these inhibitors are also active against the medically relevant stage of *T*. *cruzi*, intracellular amastigotes, suggesting that TcSir2rp1 may be a viable drug target to explore for the chemotherapeutic control of Chagas disease. In addition, the *in vivo* activity of the most potent inhibitor and selective compound towards *T*. *cruzi* was evaluated by bioluminescence imaging.

## Methods

### Ethics statement

All experiments involving animals were carried out in accordance with the IBMC.INEB Animal Ethics Committees and the Portuguese National Authorities for Animal Health guidelines, according to the statements on the directive 2010/63/EU of the European Parliament and of the Council.BPC and ACdS are accredited for animal research (Portuguese Veterinary Direction—DGAV, Ministerial Directive 113/2013). DGAV approved the animal experimentation presented in this manuscript under the license number 0421/000/000/2013, from 1i3S–Instituto de Investigação e Inovação em Saúde, Universidade do Porto, Porto, Portugal; 2IBMC-Instituto de Biologia Molecular e Celular, Parasite Disease Group, Porto, Portugal.

### Cells and parasites

Mouse myoblast C2C12 cell line (ATCC) and green monkey kidney epithelial Vero cells (ATCC) were cultured with high glucose DMEM, supplemented with 10% FBS (Gibco), 25 mM HEPES, 2 mM glutamine, 100 U/mL penicillin, 100 U/mL streptomycin and maintained in a humid 5% CO_2_ atmosphere at 37°C.

*T*. *cruzi* epimastigotes of the strain CL-Brener were maintained in the logarithmic phase of growth (1.0 x 10^6^–1.0 x 10^7^ cells / mL) at 28°C in RTH/FCS medium (RPMI 1640 supplemented with trypticase, haemin, HEPES, and 10% FBS (Invitrogen)). For the generation of transgenic cell lines, 15 μg of the constructs were digested overnight (*Spe*I for the pTcINDEX construct; *NotI* for the knockout constructs), precipitated with sodium acetate / ethanol, and introduced into 1.5 x 10^7^ epimastigote cells in the logarithmic phase of growth via transfection using an AMAXA Nucleofector II device (program V-33) with the Human T-cell nucleofector kit (Lonza). Parasites were suspended in fresh growth medium and incubated for 24-hrs to allow for the expression of drug selectable markers, prior to the addition of appropriate antibiotic (Blasticidin S (Thermo Fisher Scientific; 10 μg/mL); Hygromycin B (Formedium; 100 μg/mL); and Puromycin (Calbiochem; 5 μg/mL). Cells were then incubated for a further four- to six-weeks to allow for the selection of transgenic parasites and then genetically verified by PCR using ORF and drug selection primers.

*T*. *cruzi* wild-type (wt) and luciferase-expressing (Luc^+^) Y strain trypomastigotes (made in house) were maintained by *in vitro* infection of a monolayer of Vero cells and harvested by collection of the supernatant after 5 to 7 days. The trypomastigotes were used for the *in vitro* screening and to re-infect new monolayers for up to 10 cycles, after which point the cultures were rejected and restarted from a new frozen stock. Luminescent Y strain parasites were obtained by transfection as previously described [47]. Briefly, 10 μg of the overexpression plasmid pTREX [48], containing the sequence for firefly luciferase, were transfected into logarithmic Y strain as described above. Twenty-four hours after transfection, 75 μg/mL of G418 was added to the culture and parasites were selected for 6 weeks. Epimastigotes cultures were allowed to enrich for metacyclic trypomastigotes for 3 weeks, after which the parasites were used to infect monolayers of Vero cells in the presence of G418.

### Generation of ectopic / overexpression and knockout constructs

The TcSir2rp1 open reading frame (TriTryp accession number TcCLB.508207.150) was amplified from CL-Brener genomic DNA using KOD polymerase (Novagen). The resulting PCR product encoding the full-length ORF was cloned directly into the *T*. *cruzi* expression vector, pTcINDEX [49], using *Not*I and *BamH*I restriction sites.

To construct gene replacement cassettes, the 5’ and 3’ UTR regions adjacent to the TcSir2rp1 ORF (~500 bp) were PCR amplified from genomic DNA (S1 Fig for sequences), stitched together in a ‘knitting’ PCR reaction before ligating into the pGEM-5Zf(+) vector (Promega) via *Not*I restriction sites. Following this, the antibiotic resistance markers blasticidin-S (*BSD*) and puromycin *N*-acetyltransferase (*PAC*) were cloned into the stitch sequence *BamH*I / *HindIII* sites located between the adjoining UTRs. In order to validate the genetically modified *T*. *cruzi* cell-lines generated by previously described gene knock -in / -out experiments and further determine the relative gene expression levels of TcSir2rp1; a quantitative reverse transcription-PCR (qRT-PCR) study was performed. qPCR primers were designed, and initially normalized against two constitutively expressed, previously published and verified *T*. *cruzi* housekeeping / reference genes: histone H2B (TcCLB.511635.10) of the core nucleosome structure and glyceraldehyde-3-phosphate dehydrogenase (TcCLB.506885.413). DNase treated RNA was used as the template for a two-step reverse transcription reaction to generate complementary DNA, which in turn was utilized in a quantitative PCR reaction with detection through SYBR Green dye fluorescence.

### Cloning, expression and purification of recombinant TcSir2rp1

The full coding sequence of TcSir2rp1 was PCR amplified using the primers 5’-CCATGGGAATGAATCAAGATAACGCCAACTTT-3’ and 5’-CTCGAGTTTTCGGTCTGTCTGTGTGTACATG-3’ and ligate into pET-28a (Novagen) using the *Nco*I and *Xho*I restriction sites. Recombinant expression of the C-terminal His-tagged protein was achieved in BL21 (DE3) by induction with 0.5 mM IPTG (isopropyl-β-D-thiogalactopyranoside) overnight at 18°C. Pelleted cells were suspended in 500 mM NaCl, 20 mM Tris.HCl, pH 7.6 and disrupted by probe sonication and cleared by centrifugation. The Ni^2+^-NTA purified protein was buffer exchanged to PBS via a PD-10 desalting column (GE Healthcare) and aliquots were stored at -70°C prior to use for enzymatic assays. Protein was Western blotted using a rabbit polyclonal anti-HisTag antibody in a dilution of 1:1000. For crystallography, the full length TcSir2rp1 (M1-K359) was cloned into an in-house modified pET-28b vector, where the thrombin cleavage site is replaced by a tobacco etch virus (TEV) cleavage site and verified by sequencing. Expression of TcSir2rp1 was performed in BL21(DE3) in auto induction medium at 18°C overnight. Spun down bacterial cells were suspended in lysis buffer (50 mM Tris.HCl (pH 7.5), 500 mM NaCl, 1 mM dithiotreitol (DTT), 2mM n-octyl β-D-glucopyranoside (n-OG) and 20 mM imidazole supplemented with protease inhibitor cocktail. Soluble tagged protein was recovered from the supernatant following centrifugation of the cell homogenate by affinity chromatography on His60 Ni Superflow Resin (Clontech). Lysis buffer was used to wash the resin and protein was eluted by buffer A (50 mM Tris.HCl (pH7.5), 500 mM NaCl, 1 mM DTT, 2 mM n-OG and 500 mM imidazole). Recombinant TEV was used to remove the tag (overnight incubation at 4°C). The second purification step consisted of anion exchange chromatography (HiTrap Q HP 1 mL) with an increasing NaCl gradient in 50 mM Tris.HCl (pH 7.5), 2 mM n-OG and 1 mM DTT. Protein eluted at around 150 mM NaCl. Fractions containing pure protein were pooled and the protein was concentrated to ~9 mg/mL.

*T*. *brucei* Sir2rp1 (accession number: AAX70528.1) tagged with a C-terminal hexahistidine was expressed and characterized essentiality as described previously [50] *L*. *infantum* Sir2rp1 was expressed and purified as previously described [22].

### Deacetylation assays

The deacetylase activity was measured using the commercial SIRT1/Sir2 Deacetylase Fluorometric Assay Kit (CycLex,Japan) according to the manufacturer’s recommendations. Briefly, fluorescence emission after digestion of deacetylated substrates by a lysine specific endopeptidase was measured in a fluorometric plate reader (Synergy HT, BioTek) with excitation and emission wavelengths set at 340 and 440 nm, respectively, every 30 seconds for 1 hour. The slope of the linear part of the reaction was calculated and used as readout of enzyme activity.

Kinetic constants were determined with 0.5 μg of TcSir2rp1. For the determination of the peptide substrate constants, NAD^+^ concentration was fixed at an excess of 2000 μM while the peptide substrate concentration was varied between 0.63 and 40 μM. For the NAD^+^ constants, the peptide substrate concentration was kept in excess at 40 μM while NAD^+^ concentration was varied from 15.63 to 2000 μM. Initial velocities were measured between 4 and 6 minutes where steady state conditions were assumed. Data was analysed with GraphPad Prism version 6.0 software using the built-in enzyme kinetics, Michaelis-Menten equation regression for *K*_m_ and *V*_max_ determinations. *k*_cat_ was calculated from the total enzyme concentration [E]_t_ according to the formula in

$$k_{cat}=\frac{V_{max}}{{\left[E\right]}_{t}}$$ Eq 1

Potential inhibitors, including newly synthesized BNIP derivatives, were tested using 200 μM NAD^+^ and 10 μM of peptide substrate as previously described [46]. The inhibition is expressed in percentage and was calculated as the ratio of velocity for the linear portion of the reaction, normalized with a no drug control and a reference drug control (nicotinamide at 2 mM).

### Synthesis of BNIP derivatives

The following compounds were synthesized as previously reported **1–11** [46,51], **6b** and **9a** [52], and **6c** [53]. Compounds **1a-c, 6a, 7a 12** and **13** are newly synthetized in this study, see supporting information file for details.

### Screening of enzyme inhibitors

A variety of the bisnaphthalimidopropyl (BNIPs) derivatives, as well as the newly synthetized derivatives were evaluated as potential TcSir2rp1 inhibitors. The enzymatic reactions were performed using a commercially available CycLex SIRT1/Sir2 deacetylase fluorimetric kit (CycLex Co. Ltd., Nagano, Japan) in the absence and presence of the various inhibitors, 200 μM NAD^+^ and 10 μM of peptide substrate as previously described [46]. The inhibition is expressed in percentage and was calculated as the ratio of velocity for the linear portion of the reaction, normalized with a no drug control and a reference drug control (nicotinamide at 2 mM).

### Crystallization, data collection and processing

Prior to crystallization, purified TcSir2rp1 was incubated with three molar equivalents of acetylated p53 peptide (372-KKGQSTSRHK-K[Ac]-LMFKTEG-389). Co-crystallization experiments were carried out using the sitting drop vapor diffusion method in 96-well plates using an Innovadyne nanodrop robot. Crystals were grown in drops composed of 300 nL protein/p53 mixture and 300 nL crystallization condition (100 mM MES pH 6.5 and 1.6 M MgSO_4_) at 4°C. Crystals were flash-frozen in liquid nitrogen in crystallization condition supplemented with 25% glycerol. Diffraction data were collected on Proxima 2 beamline (SOLEIL, Saclay, France) on a Dectris Eiger 9M detector. Data were processed with xds [54] and CCP4 software package [55].

### TcSir2rp1 protein structural model

An initial TcSir2rp1 structure was obtained by molecular replacement with MrBump [56] using an “in-house” determined LiSir2rp1 structure (pdb: 5Ol0) (Ronin *et al* manuscript submitted). Refinement of the TcSir2rp1 model was done by iterative cycles of building with coot [57] and refinement with refmac [58]. The TcSir2rp1 3.5 Å structure consists of Chain A = TcSir2rp1: H10-I56 + Y63-T251 + C299-G332 + 1 Zinc ion and Chain B = Peptide p53: R_379_HKK(Ac)LMFK_386_. (SPG = P6_3_22, R_cryst_ = 21.13% / R_free_ = 28.05%, global high B value ≈ 112 Å^2^).

### Molecular docking studies

The 3.5 Å crystal structure of TcSir2rp1 was used for docking of compounds **9, 1a** and **1b**. Additionally, crystal structures of human SIRT2 were downloaded from the RCSB Protein Data Bank [59] (3zgo, [60]; 4rmj, [61]; 4rmg, [62]. AutoDock Tools version 1.5.6 was used to prepare the protein model for docking [62]. We used the protein models with and without ligands (an acetylated fragment of p53 for TcSir2, ADP and nicotinamide for 4rmj, NAD^+^ and SirtReal for 4rmg). Water molecules were removed, polar hydrogens added, charges for the protein calculated and a pdbqt file for each protein model produced. Coordinates for the docking search grid were also determined. Ligands were prepared for docking by drawing their structures in Chem3D, optimising mm2, and writing the structures to pdb files. AutoDock Tools were then used to produce a PDBQT file for each ligand [63]. Docking was performed with AutoDock Vina [64]. PyMOL (version 1.5.0.4, [65]) was used to visualize the molecular models.

### Anti-*Trypanosoma cruzi* assays

*T*. *cruzi* epimastigotes were seeded at 1.25 x 10^6^ cells/mL in a 96-well plate in the presence of varying concentrations of test compounds. Following a 64-hour incubation, 15 μL of a resazurin solution (Sigma-Aldrich; 1.1 mg/mL in PBS). Following an additional 6-hr incubation, cell viability was determined by measuring the fluorescence of each culture with a BioTek FLX800 Fluorescence Microplate Reader (excitation and emission wavelengths of 540/535 nm and 590/610 nm, respectively) and associated Gen5 Reader Control 2.0 software. EC_50_ values were determined by nonlinear regression to a sigmoidal dose-response curve using GraFit software (Version 5.0, Erithacus Software). Each assay was performed in at least triplicate in parallel to the known trypanocidal agent nifurtimox.

For the evaluation of the activity against *T*. *cruzi* amastigotes, high-content screening (HCS) was designed. In summary, 100 μL of host C2C12 cells (ATCC) were seeded in clear bottom black 96-well plates at a density of 2.5 x 10^4^ cells/mL (2.5 × 10^3^ cells/well). After 24 hrs, 50 μL of Y strain tissue culture-derived trypomastigotes were used to infect the host cells at a density of 7.5 x 10^5^/mL (3.75 x 10^4^ parasites/well). The parasites were allowed to infect for a 24 hr period, after which compounds were added in a volume of 50 μL. Final concentration of DMSO in the assay did not exceeded 0.5%. After 72 hrs of compound incubation, the wells were fixed with 4% paraformaldehyde for 15 to 30 mins. The plates were then washed once with deionized water and stained with a solution of DAPI (3 μM) for 1 hour. Plates were imaged in an INCell 2000 (GE Healthcare) high-content analyser by taking 16 pictures per well at 20 X objective amplification. Images were analysed with INCell Developer software (GE Healthcare) using the segmentation of host cell nuclei and parasite kinetoplast DNA. The measurement output used was the average number of parasites per cell calculated by the ratio of host cell nuclei/parasite kinetoplast DNA.

### *In vitro* toxicity assays

To evaluate toxicity towards mammalian cell lines (hepatocytes (Seralab), neurons and MDCK cells (ATCC); a renal cell line from dogs), all the compounds were assayed with MTT [66]. Additionally, for cardiotoxicity evaluation, where toxic effects in cardiomyocytes could be affecting their function before being lethal, a hERG assay was performed according to the manufacturer’s protocol Predictor hERG Fluorescence Polarization Assay (Invitrogen). Briefly, inhibition of hERG channel was analysed by competition of BNIPSpd and a high-affinity red fluorescent hERG channel ligand. BNIPSpd (**9**) was mixed with the ligand and membranes containing hERG channel, incubated for 2 hrs at RT and the fluorescence polarization was measured using 530 / 590 nm ex/em filters using the Synergy 2 plate reader (Biotek).

*In vitro* assays were also performed in primary cells (hepatocytes and neurons) to evaluate BNIPSpd (**9**) potential toxicity: (a) host cell nuclei counting after HOECHST staining, (b) viability by measuring the metabolism of WST-8 probe, (c) apoptosis induction through caspase 3/7 activation, (d) evaluation of mitochondrial dysfunction by measuring the membrane potential using TMRM probe, (e) membrane integrity by measuring the extracellular LDH, (f) DNA damage by histone H2AX phosphorylation (exclusively in hepatocytes) and (g) imaging of neurite outgrowth (exclusively in neurons).

The assays were performed 24 hrs after cell collection and isolation. For apoptosis, TMRM, HOECHST and WST-8 assays, cells were seeded at a density of 5000 cells/well in coated 96-well plates, incubated for 24 hrs with the compound and then stained during 1 hour with the following fluorescent probes: 5 μM CellEvent Caspase 3/7 Green Detection Reagent (Invitrogen) for measuring caspase 3/7 activation, 50 μM tetramethyl rhodamine methyl ester (TMRM probe, Anaspec Inc.) for measuring mitochondrial depolarization related to transient cytosolic Ca^2+^ signals, 5 μg/mL of HOECHST (Sigma-Aldrich) for nuclei detection and 10 μL/well of WST-8 reagent (Sigma-Aldrich) to detect viable cells. Then, absorbance at 450 nm was measured in order to analyse cell viability (WST-8). Cells were washed three times and analysed using the automatic fluorescence microscope BD Pathway 855. Pictures were taken using a 20 X objective and 488/515 nm ex/em filters for CellEvent Caspase 3/7 reagent, 555/ 645 nm ex/em filters for TMRM and 380/460 nm ex/em filters for HOECHST. Data were analysed with AttoVision (Becton Dickinson). The LDH assay was performed according to the manufacturer's protocol, Cytotoxicity Detection Kit LDH (Roche). LDH release was measured in mU/L in culture media obtained from cells subjected to treatments for 24 hrs, by measuring the released LDH rate of oxidation of NADH to NAD^+^ at 340 nm using Synergy 2 from BioTech.

For the neurite outgrowth assay, samples were washed with PBS, fixed using methanol for 10 mins at -20°C. The fixed samples were washed three times with PBS and permeabilized with PBS-Triton X-100 0.3% for 10 mins, washed three times and then blocked with BSA 0.5% in PBS for 30 mis. Anti-tubulin III antibody (Sigma-Aldrich) was added at 1/1000 dilution in blocking solution and incubated for 60 minutes at room temperature. After three washing steps, a secondary antibody Alexa 488 was added at 1/100 and incubated for 60 mins. After washing three times with PBS, pictures were taken using the BD Pathway 855 automated fluorescent microscope at 488/515 nm ex/em. Neurite average length was calculated using the neurite outgrowth module of AttoVision software (Becton Dickinson).

DNA damage was evaluated by washing cells with PBS and fixation using paraformaldehyde 3% in PBS for 15 mins. After which they were washed three times with PBS, permeabilized with PBS-Triton X-100 0.3% for 10 mins, washed again prior to blocking with PBS-BSA 0.5% for 30 mins. H2AX antibody (Abcam) was added at 1/400 in PBS-BSA 2.5% and incubated for 60 mins at RT. After three washing steps, the secondary antibody Alexa 488 was added 1/100 and incubated for 60 mins. After washing three times with PBS, pictures were taken using the BD Pathway 855 automated fluorescent microscope at 488/515 nm ex/em filters. To determine the DNA damage, intensity in the nuclei was analyzed using AttoVision software (Becton Dickinson).

Nimesulide (400 μM) was included as a positive toxicity control, and the vehicle as a negative control. The relative percentage of deviation from the negative control was quantified and assigned with a number from 0 to 5 according to the following criteria: 0 (0–20% deviation), 1 (20–40%), 2 (40–60%), 3 (60–100%), 4 (100–1000%), or 5 (>1000% deviation). The sum of these values was further ranked to create a combined injury criteria that varied from no injury (0), low injury (1 to <5), moderate injury (≥5-to <12) to high injury (≥ 12).

### *In vivo* activity

To evaluate BNIPSpd (**9**) efficacy *in vivo*, five- to six-week-old female BALB/c mice (Charles River) and infected with *T*. *cruzi* trypomastigotes expressing luciferase. Parasites were collected from the supernatants of a monolayer of Vero infected cells, washed and suspended in PBS-glucose 0.2%, and injected intraperitoneally (1 x 10^4^ per mouse). After 7 days of infection, mice were treated with drugs for 4 consecutive days orally with (benznidazole in 20% Kolliphor HS 15) or intravenously with (BNIPSpd (**9**) in 10% DMSO) at a dose of 100 or 5 mg/kg/day, respectively. Infection and treatment efficiency were evaluated following subcutaneous injection of 2.1 mg of luciferin and through live imaging using an IVIS Lumina LT (Perkin Elmer). Images of the animals were analysed using the Living Image software (Perkin Elmer).

### Pharmacokinetics

The pharmacokinetic profile of BNIPSpd was determined following intravenous injection of 5 mg/kg in BALB/c mice. The blood was collected from tail veins after 0, 5, 15, 30, 45 min, 1, 3, 24, 48 and 72 h of drug administration. Blood concentrations of the drug were analysed by UHPLC- ESI-MS/MS.

## Results

### Genetic validation of TcSir2rp1

In order to evaluate the role of TcSir2rp1 in *T*. *cruzi* survival and infectivity, a genetic validation approach was taken. Multiple attempts to knockout a single allele in epimastigotes (with various different constructs/drug selection markers) of TcSir2rp1 failed, indicating a possible lack of mono-allelic viability and thus essentiality. As such, a non-epitope tagged ectopic copy of the gene was successfully introduced into epimastigotes on a pTcINDEX over-expression vector, principally to introduce an ectopic copy of the gene to *T*. *cruzi*, and thus did not require any degree of control over the level gene expression. This was followed by successful sequential knockout of both wild-type alleles as confirmed by PCR (S2 Fig), qRT-PCR, revealed that introduction of the pTcINDEX increased the RNA level for TcSir2rp1 by ~30-fold compared to wild-type. The growth and morphology of all of these genetically altered cell-lines were indistinguishable from wild-type cells. These data strongly suggest that TcSir2rp1 is an essential gene in epimastigote *T*. *cruzi*.

### TcSir2rp1 is a canonical sirtuin with NAD^+^-dependent deacetylase activity

**Fig 1:**
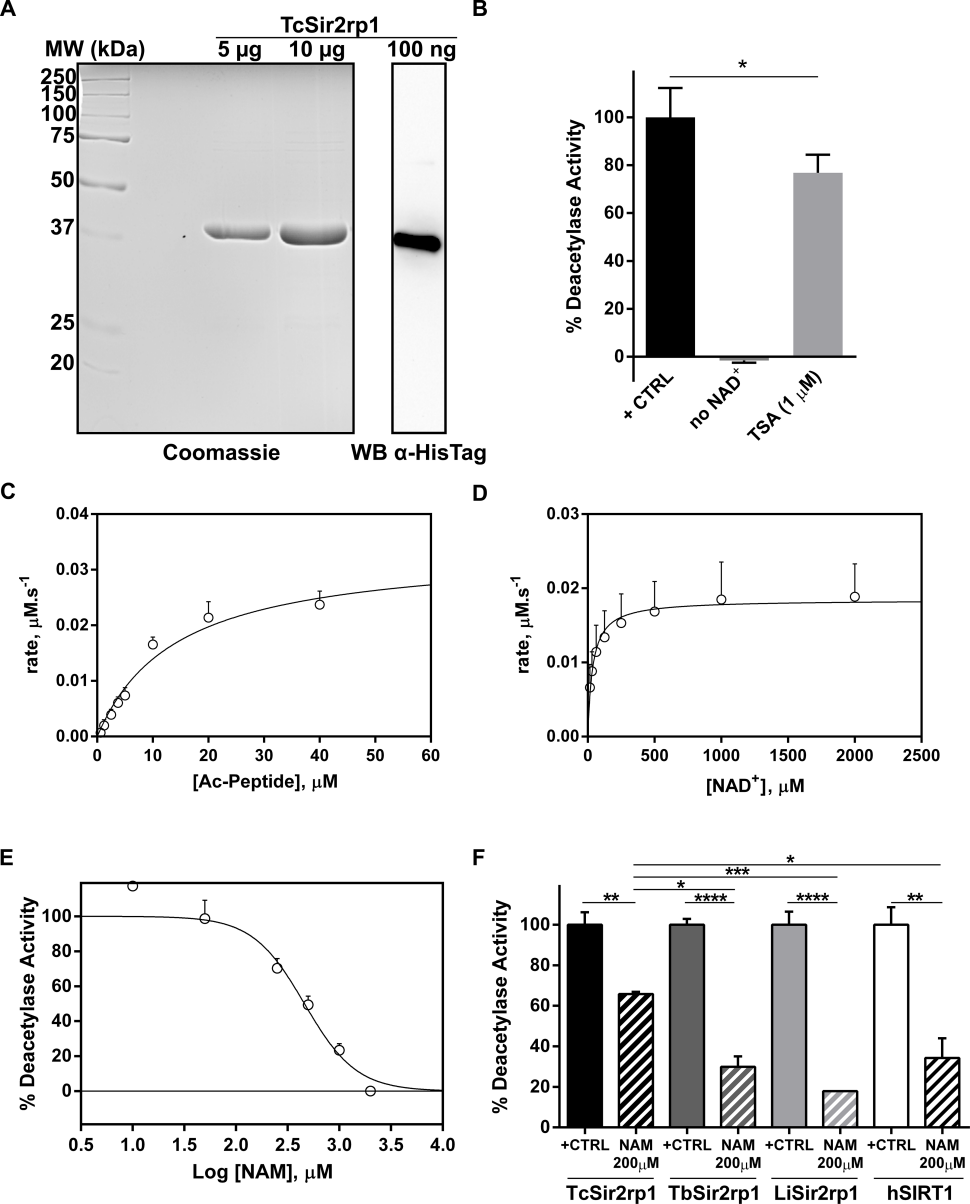
*Trypanosoma cruzi* Sir2rp1 characterization and inhibition by nicotinamide. **A)** Purity analysis of 5 and 10 μg of TcSir2rp1 by SDS-PAGE stained with Coomassie brilliant blue (left panel). Western Blot analysis of 100 ng of the hexa-histidine tailed TcSir2rp1 recombinant protein with an anti-HisTag antibody. **B)** TcSir2rp1 deacetylase activity was measured with a fluorimetric kit in the presence or absence of NAD^+^ (+CTRL), and in presence of 1 μM of trichostatin A (TSA). Bars represent mean + standard deviation. Data from 3 independent experiments. **C)** Deacetylation reactions for the determination of the kinetic constants of Ac-peptide. NAD^+^ was fixed at 2000 μM while Ac-peptide was varied (0.63 to 40 μM). Plots of initial velocities versus [Ac-peptide] were fitted to the Michaelis-Menten equation, yielding the kinetic constants (see Table 1). **D)** Deacetylation reactions for the determination of the kinetic constants of NAD^+^. Ac-peptide was fixed at 40 μM while NAD^+^ was varied (15.63 to 2000 μM). Plots of initial velocities versus [NAD^+^] were fitted to the Michaelis-Menten equation, yielding the kinetic constants (see Table 1). Dots and error bars represent mean + standard deviation. **C-D**) Data from 3 independent experiments. **E)** Dose-response curve of TcSir2rp1 inhibition by nicotinamide. Data represents the average + standard deviation of three independent experiments. **F)** Differential inhibition of TcSir2rp1, TbSir2rp1, LiSir2rp1 and hSIRT1 by a dose of 200 μM of nicotinamide (NAM). Bars represent mean + standard deviation. Data from 2 independent experiments. Differences between the experimental groups were considered significant as follows: * p<0.05, ** p<0.005, *** p<0.001 and **** p<0.0001.

Recombinant TcSir2rp1 was expressed and affinity purified as a C-terminal hexa-histidine tagged protein. The resultant protein was shown to be pure by SDS-PAGE and of the expected size (~40 kDa) (Fig **1A**). Deacetylase activity was measured as described previously for LiSIR2rp1 [22], in a coupled reaction based on the ability of lysylendopeptidase to digest deacetylated lysine, but not acetyllysine, residues, thereby releasing a quencher group from the molecule, allowing the fluorophore to emit fluorescence in a time-resolved manner. Deacetylase activity was dependent on NAD^+^ confirming TcSir2rp1 to be a NAD^+^-dependent deacetylase (Fig **1B**). Furthermore, when incubated with the histone deacetylase class I and II inhibitor trichostatin A (TSA) at 1 μM, TcSir2rp1 still displayed 77 ± 8% of activity (Fig **1B**). TSA is a poor inhibitor of sirtuins (class III deacetylases), confirming the classification of the protein as a canonical Sir2.

The steady-state kinetic parameters (*K*_m,_ *V*_max_, *k*_cat_ and *k*_cat_/*K*_m_ values) of deacetylase activity were determined (Table 1). By way of confirmation, the same kinetic parameters were also independently assessed using an electrophoretic mobility shift assay, performed as described previously [67]. Highly similar values (Km’s of 38.7 ± 1.5 μM for NAD^+^ and 32.3 ± 6.0 μM for substrate (TSPQPKK-Ac)) were obtained using the alternative microfluidic based assay confirming that the two different techniques generated essentially similar results when used to assess TcSir2rp1 NAD^+^-dependent deacetylase activity. The kinetic data obtained for the NAD^+^ cofactor is highly similar to that obtained for *T*. *brucei* Sir2rp1 whilst the substrate Km values reported here are 2-4-fold lower [68]. When compared to yeast Sir2 and the human SIRT2, TcSir2rp1 also displays a high deacetylation activity as demonstrated by the catalytic efficiency (*K*_m_/*k*_cat_) constants calculated for the homologue enzymes [69, 70]. Catalytic efficiencies are the most relevant constant in physiological conditions since they define the rate of the reaction when substrate concentrations are not at saturating levels, as for most cellular enzymatic reactions [70].

**Table 1:** Kinetic parameters determined for TcSir2rp1.

	Ac-Peptide	NAD^+^
*K*_m_ (μM)	14.23 ± 0.61	38.32 ± 9.64
*v*_max_ (μM.s^-1^)	0.034 ± 0.003	0.018 ± 0.004
*k*_cat_ (s^-1^)	0.136 ± 0.013	0.075 ± 0.018
*k*_cat_/*K*_m_ (mM^-1^.s^-1^)	9.585	1.956

The above results were used to establish a standard screening assay (10 μM Ac-peptide and 200 μM NAD^+^). Nicotinamide was tested in order to evaluate if small molecules could inhibit the enzymatic activity described. Nicotinamide is a classic non-competitive inhibitor of sirtuins that has been used to characterize several enzymes [22, 71]. A dose-response curve of nicotinamide inhibition of TcSir2rp1 indicates an IC_50_ of 456 ± 44 μM (Fig 1E). This value is significantly higher when compared to the IC_50_ determined for other sirtuins like the human SIRT1 (118.3 ± 23.6 μM), the *Plasmodium falciparum* Sir2A (51.2 ± 3.0 μM) or the LiSir2rp1 (39.4 ± 5.0 μM) previously described [46, 72]. When a concentration of 200 μM of nicotinamide was tested against TbSir2rp1, LiSir2rp1 and the human SIRT1, TcSir2rp1 only had a mild inhibition of 34 ± 1%, whereas the inhibition was 70 ± 5%, 82 ± 0%, 66 ± 10% for TbSir2rp1, LiSir2rp1 and human SIRT1, respectively (Fig 1F). Some studies have previously reported the anti-parasitic activity of nicotinamide on *T*. *cruzi*, but, to our best knowledge, this is the first report that proves the molecular inhibition of the parasite sirtuin by nicotinamide [45, 73].

### Inhibitory activity of BNIP derivatives on TcSir2rp1

**Fig 2:**
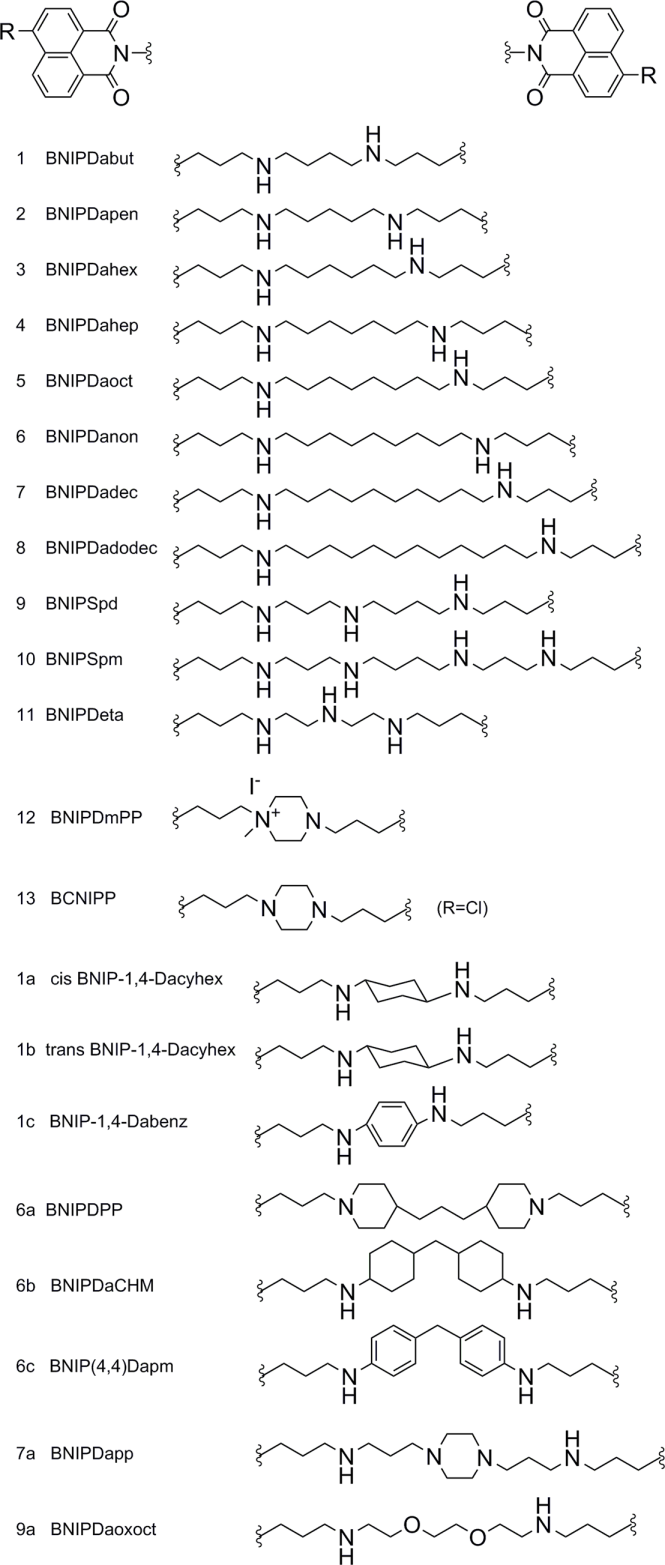
Chemical structures of the bisnaphthalimidopropyl derivatives used in the present study. R = H in all compounds except for compound 13.

**Fig 3:**
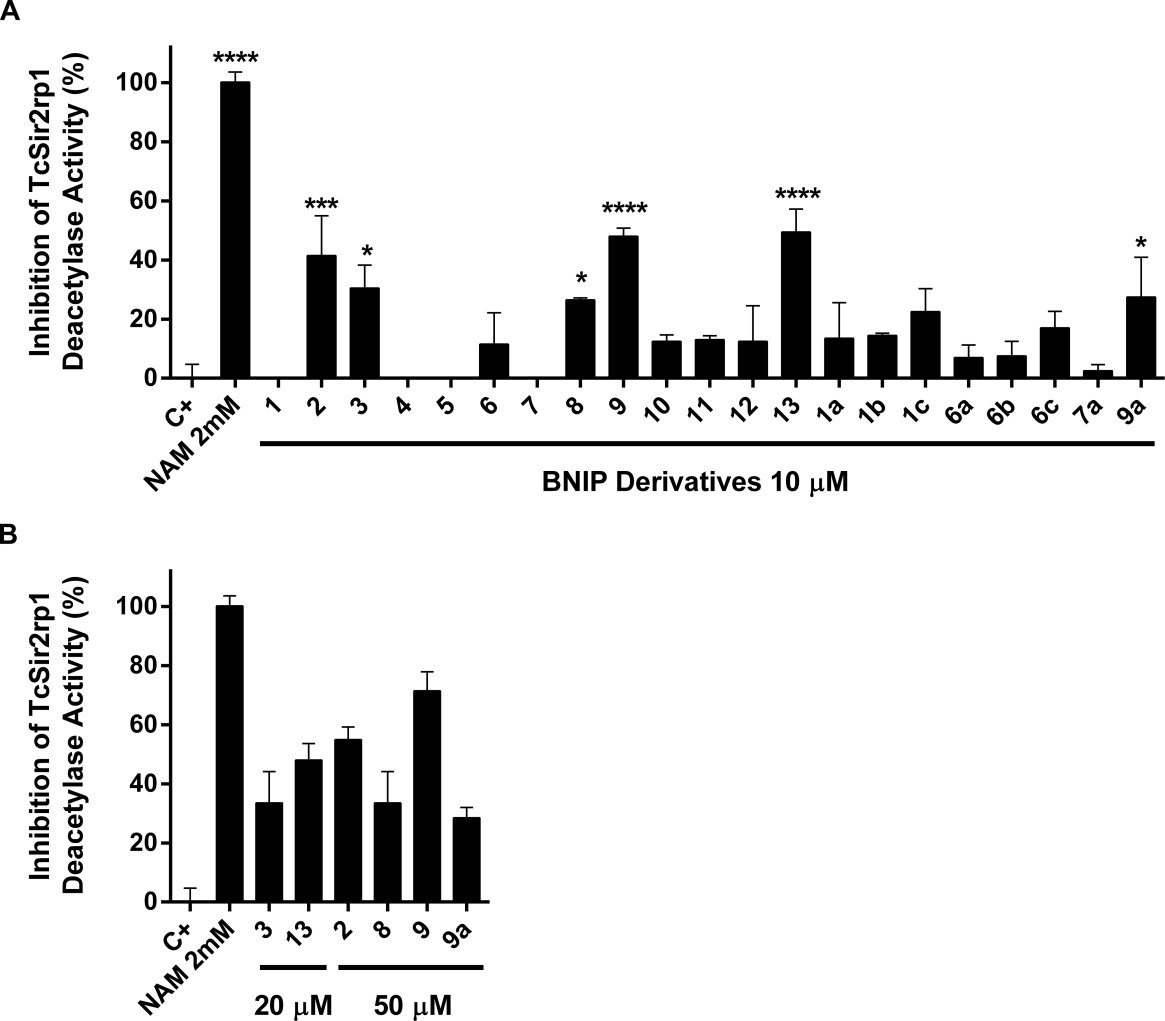
Enzymatic inhibition of TcSir2rp1 by BNIP derivatives. **A-B)** The percentage of inhibition of the NAD^+^-dependent deacetylase activity of TcSir2rp1 by BNIP derivatives is represented in the y axis. Positive (no drug) and negative (NAM, nicotinamide at 2 mM) controls are represented. Bars represent the average + standard deviation of at least two independent experiments. Differences between the experimental groups were considered significant as follows: * p<0.05, *** p<0.001 and **** p<0.0001.

We next evaluated the enzymatic inhibition of TcSir2rp1 by a class of experimental compounds previously characterized as inhibitors of the orthologue enzyme of *L*. *infantum* [46] as well as the newly synthetized derivatives. BNIP derivatives are constituted by two naphthalimidopropyl groups separated by a linker that varies in length and functional groups, and have been characterized as NAD^+^-competitive inhibitors of LiSir2rp1 [74]. In this study, an additional set of compounds was synthetized with the objective of improving cellular target binding by including heteroatoms in the alkyl chain connecting the two naphthalimidopropyl groups, as well as by introducing cyclic structures in the linker (Fig 2). The results are summarized in Fig 3A and indicate the percentage of inhibition of the NAD^+^-dependent deacetylase activity for a single dose concentration of 10 μM. Of all the compounds tested, compounds **2, 3, 8, 9, 13** and **9a** had a statistically significant inhibition of the enzyme. In order to verify a concentration dependent effect on enzymatic inhibition, compounds **2, 8, 9** and **9a** were additionally tested at 50 μM, whereas compound **3** and **13** were tested at 20 μM due to low solubility. Inhibition percentages for this higher concentration were 55 ± 4%, 34 ± 11%, 31 ± 9%, 72 ± 6%, 48 ± 6 and 29 ± 4% for compounds **2, 3, 8, 9, 13** and **9a**, respectively (Fig 3B). Only compound 9 demonstrated a statistically significant dose-dependent increase in inhibition (1-way ANOVA, p value <0.05). In fact, compound **9** was one of the most active compounds against TcSir2rp1, with a 10 μM dose inhibiting almost 50% of the NAD^+^-dependent deacetylase activity, a concentration 45 times lower than the necessary for nicotinamide to achieve the same level of inhibition. Compound **9** is a derivative of compound **5** by substitution of one of the carbons in the linker chain by a nitrogen atom. Since compound **5** was not active against the enzyme, it is possible that the substitution of the nitrogen allows the establishment of additional molecular interactions with the enzyme and/or modifies the rigidity of the linker chain resulting in higher inhibition. Compound **9a** is also a derivative from compound **5** with eight atoms in the linker chain separating the two naphthalimidopropyl groups, but with two oxygen atoms in substitution of two carbons. This modification also seems to increase the inhibitory activity against TcSir2rp1 (28 ± 13%), but it is not as marked as for compound **9**. Although a clear correlation between the length of the linking chain and enzymatic inhibition was established for the *L*. *infantum* orthologue [46], the same was not verified with TcSir2rp1. Compounds with 5 and 6 carbons in the linker chain also displayed some inhibitory activity against the enzyme (compound **2**, 42 ± 13% and **3**, 31 ± 8%, respectively), but when tested at a higher concentration, the increase was not statistically significant. Compound **3** was only tested at 20 μM due to poor solubility. In general, the most active inhibitors of TcSir2rp1 were also selective towards the *T*. *cruzi* enzyme, as demonstrated by inhibition of a human sirtuin homologue, hSIRT1. In addition to hSIRT1 inhibition previously described for compounds **1–11** [46], compounds **12-9a** were also tested at 10 μM against this enzyme (S1 Table, see supporting information). Compound **6b** was the most active against the human homologue enzyme (44 ± 8% of inhibition), but was a weak inhibitor of TcSir2rp1 (8 ± 5%) at the same concentration of 10 μM. Compound **13** also had a reduced activity against hSIRT1 (15 ± 4%), but was a much stronger inhibitor of the *T*. *cruzi* enzyme, with an inhibition at least 3-fold higher (50 ± 8%).

### BNIPs are potent on-target *in vitro* inhibitors of *Trypanosoma cruzi* epimastigotes

As the deacetylase activity was inhibited by some BNIP derivatives, they were also tested against *T*. *cruzi* epimastigotes (Table 2). Some of these compounds (**3**, **6** and **9**) showed moderate activity (EC_50_), while compounds **7** and **2** were just as potent as nifurtimox, the positive control. To investigate if the BNIP derivatives’ trypanocidal activity is correlated with Sir2rp1 inhibition, compounds were also tested against the pTcINDEX overexpressing TcSir2rp1 cell-line, described earlier. As expected the nifurtimox EC_50_ did not alter significantly compared to the wild-type cell line, nor did that of compound **7**. However, the EC_50_ values of the other 4 BNIP derivatives did increase 2.8–4.8 fold, strongly suggesting that these BNIP derivatives were trypanocidal, because they were “on-target”, i.e. inhibiting TcSir2rp1and causing cell death.

**Table 2:** EC_50_ values (μM) of BNIP derivatives and nifurtimox against *T*. *cruzi* epimastigotes, wild-type (WT) and cells overexpressing TcSir2rp1 (OE) with the pTcINDEX.

Compounds	WT	OE	Fold-increase
2—BNIPDapen	1.77 ± 0.68	7.94 ± 0.78	4.49
3—BNIPDahex	33.6 ± 1.5	102.3 ± 9.9	3.04
6—BNIPDanon	23.4 ± 2.8	112.6 ± 8.2	4.81
7—BNIPDadec	2.53 ± 0.41	2.97 ± 0.60	1.17
9—BNIPSpd	13.3 ± 0.6	36,1 ± 1.5	2.77
Nifurtimox	2.11 ± 0.15	2.24 ± 0.14	1.06

Values are means ± SD (n = 4)

### BNIPs are potent *in vitro* inhibitors of *Trypanosoma cruzi* intracellular amastigotes

Once demonstrated that the deacetylase enzymatic activity is inhibited by some BNIP derivatives and they are also on-target against epimastigotes, the compounds were next tested using an *in vitro* HCS assay on the medically relevant intracellular form of amastigotes (S3 Fig). In this assay, the compounds are incubated for 72 hours with cells previously infected with wild-type trypomastigotes for 24 hours. The readout was done by comparing the average number of amastigotes per cell that developed by the end of the incubation period. The quality of the assay was statistically evaluated by the calculation of the z-factor [75], that represents the reliability of the assay in distinguishing positive controls confidently from negative controls. Varying from 0 to 1, z-factor values higher than 0.5 are considered acceptable for drug screening. The z-factor obtained was 0.68, validating the use of the assay (S3B Fig). The reference drug benznidazole was assayed for quality control and showed an EC_50_ of 1.23 ± 0.30 μM, in accordance with previous studies [76].

**Fig 4:**
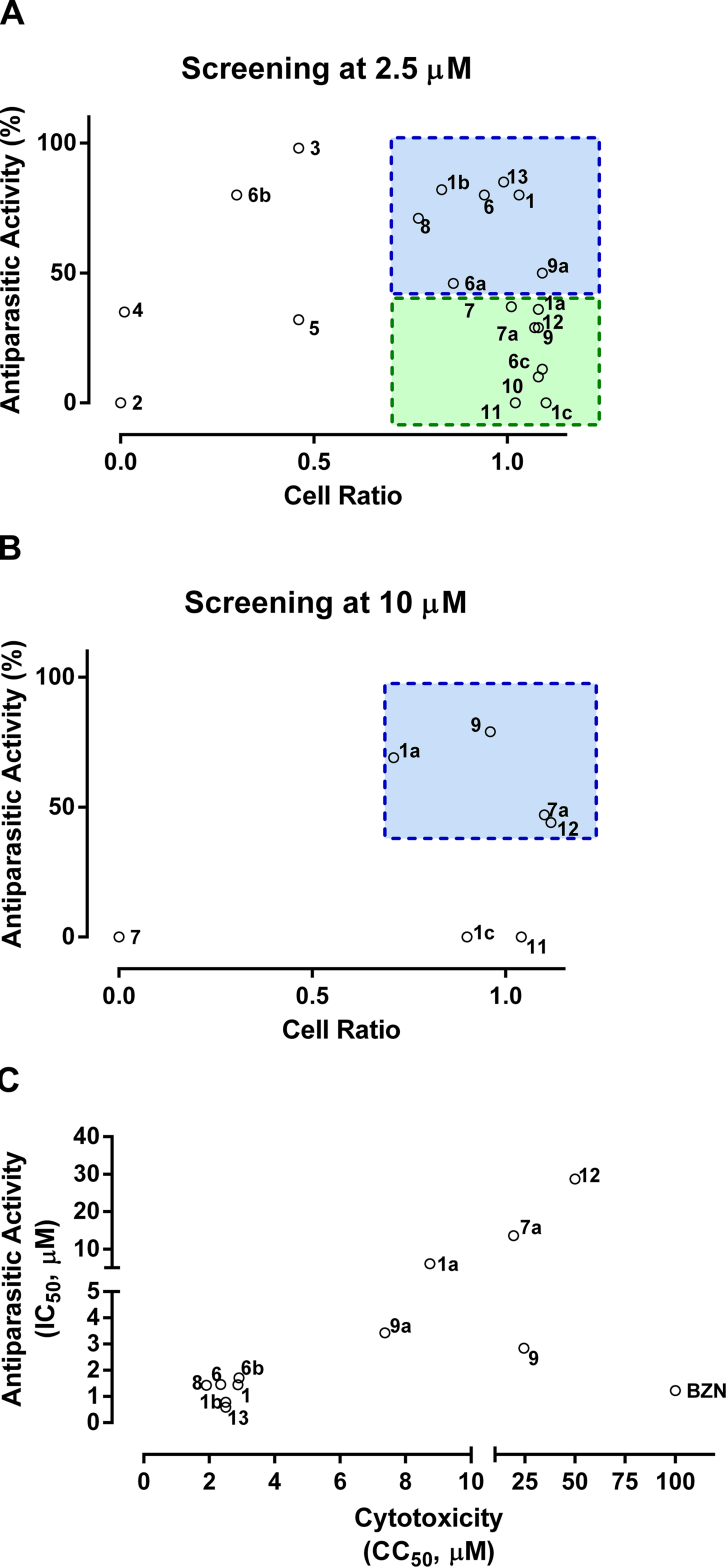
*In vitro* activity of BNIP derivatives. **A)** Primary screening of BNIP derivatives by an *in vitro* assay for intracellular *T*. *cruzi* amastigotes at a single dose of 2.5 μM. Values for both anti-parasitic activity and cell ratio are represented in the dot plot graph. The blue square represents the zone of selection for active, low toxicity hits to be evaluated by dose-response curve analysis. The green square represents the zone of selection for compounds to be tested at a higher dose. **B)** Primary screening of the compounds selected for testing at a higher dose of 10 μM. Values for both anti-parasitic activity and cell ratio are represented in the dot plot graph. The blue square represents the zone of selection for active, low toxicity hits to be evaluated by dose-response curve analysis. **C)** Representation of the selectivity indexes of the compounds analysed by dose-response curve analysis, with the determined anti-parasitic activity (EC_50_) in the y axis, and the cell ratio (CC_50_) in the x axis. Dots represent the average of three independent experiments.

**Fig 5:**
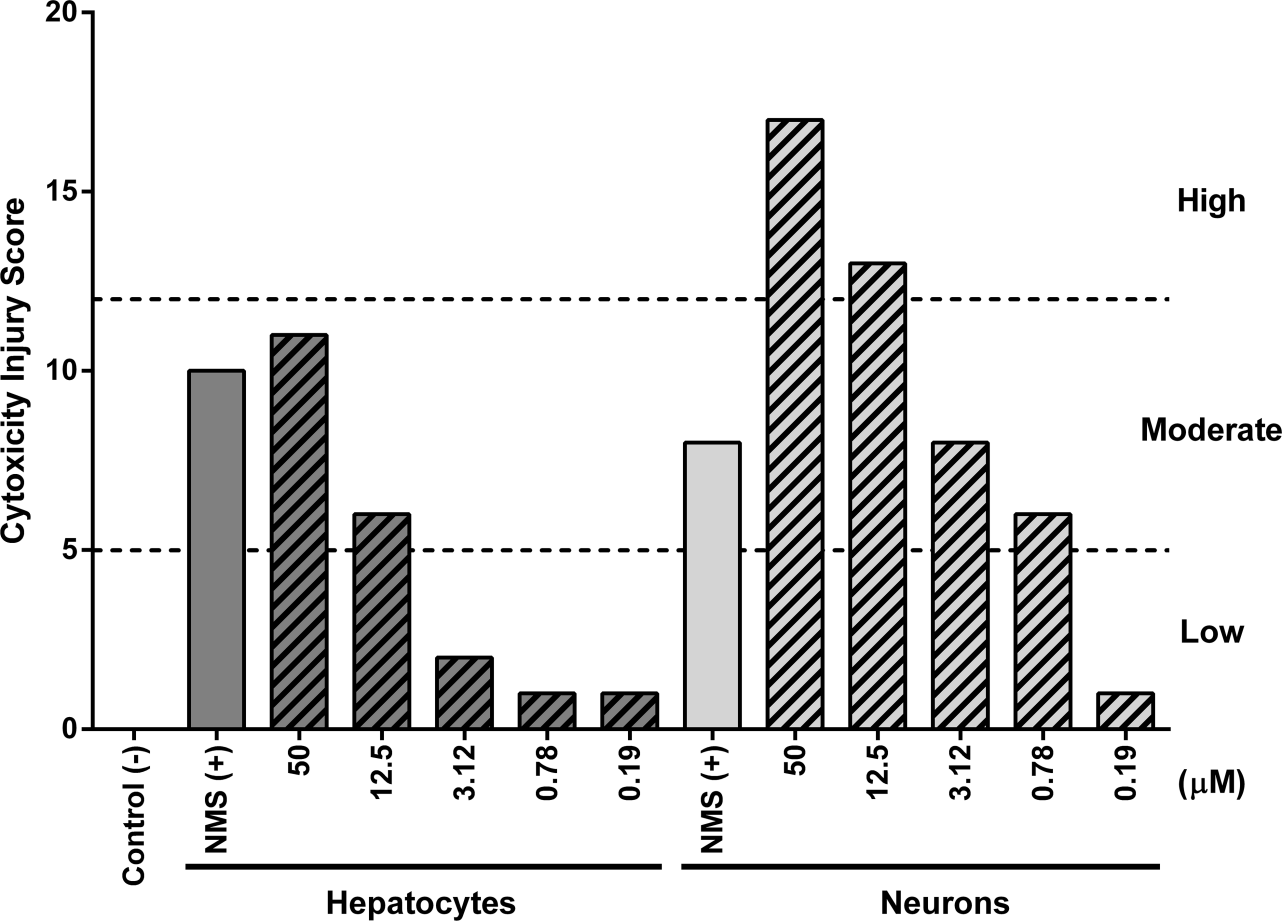
*In vitro* hepatocytes and neurons injury scores for BNIPSpd (9) by HCS. The score was calculated as the sum of individual scores obtained from a panel of *in vitro* cytotoxicity assays that include: mitochondrial dysfunction measured by TMRM probe dynamics in cells; membrane integrity assayed by lactate dehydrogenase quantification; DNA damage by imaging with H2AX antibody; apoptosis by caspase 3/7 activation; and neurite outgrowth as imaged with an anti-tubulin III antibody. Nimesulide (400 μM), an approved drug with a mild toxicological profile, was included as a toxicity control.

In order to prioritize compounds, all the BNIP derivatives were initially screened at a single dose of 2.5 μM (Fig 4A), where all of the compounds were fully soluble. The compounds that presented high anti-parasitic activity (>40%) and low toxicity [high cell ratio, defined as (average number of cells for the compound / average number of cell for the DMSO 0,5% control), >70%], represented in a blue box, were selected for dose-response curve analysis. A set of compounds that was not toxic at 2.5 μM (green box) was further tested at 10 μM. It is worth noting that compound **2,** which showed moderate inhibition of TcSir2rp1 and good on-target trypanocidal activity against *T*. *cruzi* epimastigote cells (Table 2), failed this hurdle due to high cytotoxicity issues (Fig 5A). Again, the compounds that had high anti-parasitic activity and low toxicity were selected for dose-response analysis (blue box, Fig 4B). Compounds **10** and **6c** were not tested at 10 μM due to poor solubility. Compounds **1, 6, 8, 9, 12, 13, 1a, 1b, 6a, 7a** and **9a** (Fig 4C and Table 3) were assayed by dose-response curves in the same conditions of the primary screening. Compound **9,** the strongest inhibitor of TcSir2rp1 and for on-target killing of epimastigotes (Table 2), was also active against *T*. *cruzi* amastigotes, presenting an EC_50_ of 2.84 ± 0.30 μM, in the range of the reference drug benznidazole (1.23 ± 0.30 μM). In addition, it was the least toxic of the compounds analysed by dose-response curve, with a CC_50_ of 24.47 ± 0.45 μM, resulting in a selectivity index (SI) of 8.8 units. Compound **9a**, an analogue of **9** with two oxygen atoms in the linking chain instead of one nitrogen atom, was also active and presented a similar EC_50_ of 3.43 ± 0.57 μM. However, it was less selective than compound **9** (2.1 units). Compound **5** which is also the same length between the naphthalimidopropyl groups, but only made of methylene moieties, has very little if any activity against either the enzyme or amastigotes. Compound **13**, also an inhibitor of TcSir2rp1 (50 ± 8%), was the most active compound against *T*. *cruzi* amastigotes, with an EC_50_ of 0.59 ± 0.23 μM. It was not possible to calculate the CC_50_ since the highest concentration tested allowed by the solubility of the compound (2.5 μM) did not reduce the cell ratio below 50%, making the SI of the compound at least 4.2. The second most potent hit, compound **1b**, is a derivative of compound **1** where a cyclohexane moiety was introduced as a spacer between the naphthalimidopropyl groups. The synthesis of this compound resulted in two isomers with a significant difference in their anti-parasitic activity, where the *trans* isomer (compound **1b**) presents an EC_50_ of 0.78 ± 0.12 μM and the *cis* isomer (compound **1a**) an EC_50_ of 6.09 ± 0.14 μM. Such a difference suggests this particular stereoisomeric configuration improves anti-parasitic activity. Activity of nicotinamide in the amastigote assay was also assessed, but there was no activity detected up to a concentration of 2000 μM.

**Table 3:** Anti-parasitic activity, cytotoxicity and selectivity index of BNIP derivatives selected for dose-response curve analysis.

Compound	Anti-*T*. *cruzi* Activity Amastigotes μM (EC_50_ ± SD)	C2C12 Cytotoxicity μM (CC_50_ ± SD)	Selectivity Index*
1—BNIPDabut	1.71 ± 0.23	2.91 ± 0.94	1.7
6—BNIPDanon	1.45 ± 0.37	2.35 ± 0.77	1.7
8—BNIPDadodec	1.43 ± 0.10	1.91 ± 0.06	1.3
9—BNIPSpd	2.84 ± 0.18	24.47 ± 0.45	8.8
12—BNIPDmPP	28.74 ± 4.09	> 50	> 1.7
13—BCNIPP	0.59 ± 0.23	> 2.50	> 4.2
1a - cis BNIP-1,4-Dacyhex	6.09 ± 0.14	8.74 ± 1.14	1.4
1b - trans BNIP-1,4-Dacyhex	0.78 ± 0.12	> 2.50	> 3.2
6a - BNIPDPP	1.45 ± 0.30	2.88 ± 0.12	2.0
7a - BNIPDapp	13.58 ± 2.90	19.30 ± 5.64	1.4
9a - BNIPDaoxoct	3.43 ± 0.57	7.37 ± 0.65	2.1
Benznidazole	1.23 ± 0.30	> 100	> 81.0

*Selectivity Index = CC_50_ cell line/EC_50_ *T*. *cruzi*

Values are means ± SD (n = 4)

### BNIPSpd testing in a mouse model for Chagas disease

Compound **9**, BNIPSpd, showed the best enzymatic inhibition, low EC_50s_ against epimastigotes (potentially on target) and the medically relevant amastigote stages of the parasite and some degree of selectivity. Based upon these parameters it was decided to follow up its characterization. The compound toxicity was further evaluated against a panel of primary cells to determine tissue specific toxic effects. The results of cytotoxicity against neurons, hepatocytes and MDCK cells, and the cardiotoxicity determination by hERG predictor method are summarized in Table 4.

**Table 4:** Cytotoxicity and selectivity of BNIPSpd (9).

Cell type	Cytotoxicity	Selectivity Index*
Hepatotoxicity (Hepatocytes)	≥ 3.1 μM	1.1
Neurotoxicity (Neurons)	≥ 3.9 μM	1.4
Nephotoxicity (MDCK cells)	≥ 100 μM	35.2
Cardiotoxicity (hERG predictor)	0.36 ± 0.51%	-

*Selectivity Index = CC_50_ cell line / EC_50_ *T*. *cruzi*

As shown, BNIPSpd (**9**) presents hepatotoxicity and neurotoxicity values very similar to its efficacy concentration (SI of 1.1 and 1.4 respectively). On the other hand, it seems not to be nephrotoxic (SI of 35.2) or cardiotoxic (hERG channel 99.6% functional). The toxicity values are higher than the corresponding ones for the C2C12 cells (Table 3, SI of 8.8), but this is not surprising given the fact that the primary cells are usually more sensitive. Furthermore, the toxic effects at the cellular level were evaluated and quantified by a set of *in vitro* assays that include mitochondrial dysfunction, membrane integrity, DNA damage, apoptosis and neurite outgrowth on hepatocytes and neurons (Fig 5). This analysis could not be performed in the MDCK cell line due to the high amounts of DMSO to test the required BNIPSpd (**9**) concentration being toxic for these cells.

Nimesulide, an approved and widely used anti-inflammatory drug, was included at a concentration of 400 μM as a control of toxicity [77–79]. The results are presented in Fig 5. In summary, BNIPSpd (**9**) showed a low injury score for hepatocytes, with no toxicity up to 50 μM and moderate injury score for neurons, where toxicity was most significant from 12.5 μM. These values correspond to the range within the efficacy concentration for this compound (2.84 μM). Despite showing a mild injury score for neurons, it should be kept in mind that further analysis needs to be done in order to determine whether the compound is capable of crossing the blood-brain barrier or not. Indeed, a compound could be very neurotoxic but never been able to reach the neurons. The main mechanisms responsible for the neurotoxicity are the caspase 3/7 activation, a measure of apoptosis and disruption of membrane integrity as assessed by LDH release. As the concentration of drug increases other toxicity mechanisms like mitochondrial dysfunction and neurite outgrowth start to contribute to the overall cytotoxicity score. In conclusion, up to concentrations of 10 μM, BNIPSpd (**9**) appears to be safe for further evaluation in an animal model for Chagas disease.

Finally, BNIPSpd (9) *in vivo* activity was characterized in a murine model of *T*. *cruzi* infection and using bioluminescence imaging. A bioluminescent *T*. *cruzi* Y strain (Luc+) was obtained like previously described [47] and the *in vitro* limit of detection in the bioimaging equipment IVIS Lumina LT was determined to be 10^4^ parasites (t-test, p-value < 0.05) (S4A Fig). Different numbers of Luc^+^ trypomastigotes were tested in order to assess which condition yielded the best readout (S4B Fig). When injected intraperitoneally with 10^4^ parasites, the mice developed an infection at first mostly located around the inoculation site (day 7) but that later spread to the whole body (S4B Fig). Similar observations were made for the 10^5^ and 10^6^ inoculates but 10^4^ parasites yielded a higher and more reproducible increase of the whole body bioluminescent signal.

A regimen of 5 mg/kg/day of BNIPSpd (**9**) was administered intravenously for 4 consecutive days, while a positive control with a dose of 100 mg/kg/day of benznidazole *per os* and the respective vehicle controls were also performed (S5A Fig). Unfortunately, BNIPSpd (**9**) did not exhibit any *in vivo* activity, since bioimaging analysis demonstrates the infection progressed as in the animals treated with the vehicle only, which showed on average an increase in the luminescence signal by 40-fold. By comparison, animals treated with benznidazole at 100 mg/kg/day displayed, on average, a 700-fold reduction in the bioluminescence signal compared to mice treated with vehicle only (S5A Fig).

To investigate whether the lack of anti-parasitic activity *in vivo* could be due to the pharmacokinetics profile of BNIPSpd (**9**) we proceeded to quantify the compound in the blood over time. Analysis of the snapshot pharmacokinetics profile (S5B Fig) may explain the lack of *in vivo* activity of BNIPSpd (**9**). For the first 30 mins after intravenous injection, it never reaches a concentration equivalent to the *in vitro* EC_50_ for *T*. *cruzi* amastigotes. The highest plasma concentration detected is 1.70 μM at the 5 min time-point, with the compound below the level of detection after the 3 h time-point and up to 72 h post administration (lower limit of quantification is 8.1 nM). This pharmacokinetic profile indicates that a concentration of drug able to reach and clear parasitized tissues is not achieved in this mouse model.

### TcSir2rp1 structural model and docking with BNIP derivatives

**Fig 6:**
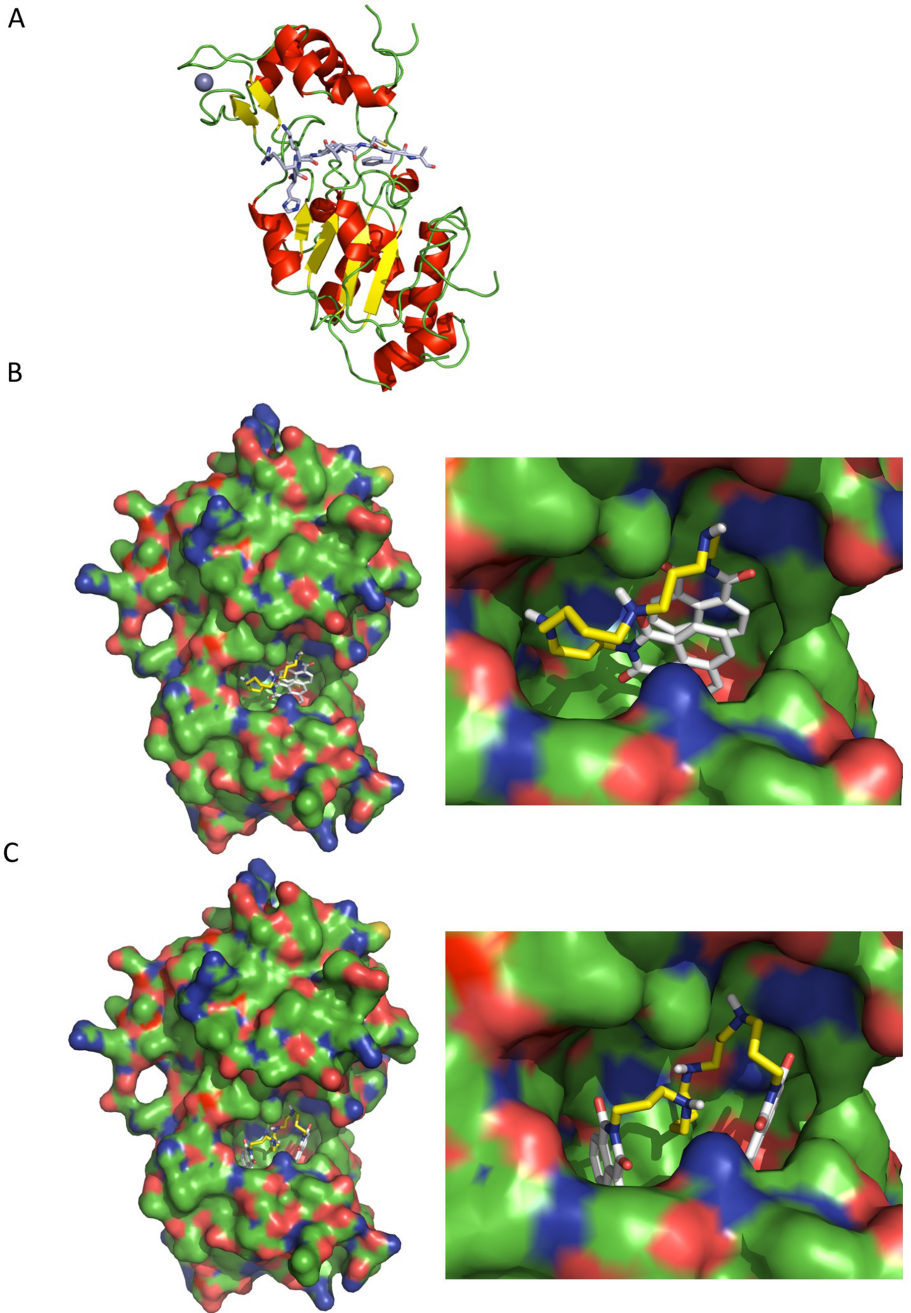
TcSir2rp1 structural model and docking with compound 9. **A**) The 3.5 Å structural model shows a large Rossmann-fold domain (composed of 6 parallel β-strands, sandwiched between 2 layers of α-helices), and a small zinc binding domain. The substrate acetylated peptide p53 is bound to the cleft between the small and the large domains. **B-C**) The substrate p53 peptide was removed from the structure and docking studies conducted with compound **9**. Several conformations (only 2 shown here) of compound **9** were possible in a putative ligand binding site close to the NAD binding site.

To understand how BNIPSpd (**9**) could interact with TcSIR2rp1, we tried to solve the X-ray structure of the protein. Unfortunately, despite extensive crystallization optimization attempts, crystals with only relative low resolution limit (3.5Å) could be obtained. Nevertheless, this allowed a preliminary TcSIR2rp1 model in complex with p53 peptide substrate to be generated. The final 3.5Å TcSir2rp1 structure (Fig 6A) shows clearly defined main chain (Cα), but only poor density is observed for side chains in some regions, thus, these side chains were removed.

TcSIR2rp1 global fold is, as expected, similar to other sirtuins including the human SIRT2 (S6 Fig) with a large Rossmann-fold domain (composed of 6 parallel β-strands, sandwiched between 2 layers of α-helices) and a small Zinc binding domain. The substrate peptide p53 is bound to the cleft between the small and the large domains (Fig 6A), the co-factor binding loop is ordered in an open conformation similar to hsSIRT2 apo structure [48] or yeast Hst2 in complex with p53 peptide and NAD [60]. D252-I298 is missing in the TcSIR2rp1 final model due to a lack of density, which suggests high flexibility and/or potential disorder in this region. Nevertheless it should not impact upon our docking experiments as not involved in active site delineation and region, located at the bottom part of the Rossmann fold domain (diametrically opposed to Zinc ion along an axis going through p53 peptide).

Docking studies were subsequently performed with the TcSir2rp1 structure, where the p53 peptide substrate was removed and compound **9** was used as a possible ligand. Several conformations of compound **9** were found in a putative ligand binding site close to the NAD binding site (Fig 6B and 6C), all with very similar binding affinities 9 ± 0.3 kcal/mol.

To investigate if this possible interaction between compound **9** and TcSir2rp1 was specific, several human SIRT2 structures were also subjected to similar docking studies, (S7 and S8 Figs), but they were found to have similar binding affinities to those observed for TcSir2rp1. Likewise, the two isomers, compound **1a** and **1b**, which showed distinct difference in EC_50_ activities (6.09 and 0.78 μM respectively) were also docked with TcSir2rp1 (S9 and S10 Figs), but they very similar binding affinities to each other.

## Discussion

Sirtuins have long been proposed as interesting targets to treat parasitic diseases [80, 81], but only recently have these proteins been characterized in *T*. *cruzi* [44, 45]. Even though the localization, expression and some of the important functions of *T*. *cruzi* sirtuins have been described, to date no biochemical characterization has been performed.

Our results demonstrate that the annotated coding sequence of TcSir2rp1 encodes for an essential (as genetically validated here) canonical sirtuin that does not display deacetylation activity in the absence of NAD^+^ and is mostly insensitive to TSA. Kinetic data obtained for TcSir2rp1 is highly similar to the values previously described for TbSir2rp1 [67]. Despite moderate differences in primary sequence (61%), a close analysis of the amino acids adjacent to the essential histidine (H142) at the catalytic site demonstrates a 100% similarity of the four amino acids upstream and downstream, which likely participate and/or help shape the catalytic site, and thus may explain the similarity observed. Nonetheless, it should be noted that distant amino acids in terms of primary structure may have an influence on substrate binding and enzymatic activity, as demonstrated in the case of PfSir2 in which the C- and N-terminal removal led to modifications in the enzyme kinetic constants and sensitivity to nicotinamide [72].

An essential step in the evaluation of a drug target is the proof of druggability. Even if a gene product is deemed essential for a given organism, if the activity or function mediated by this protein is not amenable to modulation by small molecule inhibitors, then it is not considered to be a good drug target. To this end, it was sought to modulate TcSir2rp1 with a classic inhibitor of this protein family, nicotinamide [82]. The enzymatic activity described was indeed inhibited by nicotinamide, although in concentrations higher than those previously reported for other members of the family. In particular, the IC_50_ for LiSir2rp1 and hSIRT1 is 11 and 4-fold lower, respectively, than the one reported here for TcSir2rp1 [46]. Another observation that supports the resistance to nicotinamide is the fact that even when 2 mM of NAD^+^ is used in reactions for the determination of the kinetic constants for NAD^+^, there is no decrease in enzymatic activity unlike previously reported for other enzymes [22]. Such inhibition might be expected since nicotinamide is an endogenous product of the deacetylation reaction. Nicotinamide binds to a distinct C pocket that appears to be conserved among the sirtuins that have been structurally elucidated by co-crystallography [71]. The study of the presence and conservation of this pocket in TcSir2rp1 may shed some light on the observed nicotinamide resistance. Although nicotinamide has some anti-parasitic activity against *Plasmodium* [83], *Leishmania* [84], *T*. *brucei* [85] and also *T*. *cruzi* [73], no confirmation of a sirtuin-mediated mechanism has been clearly established to date. In this study, no amastigote anti-parasitic activity was detected for nicotinamide in concentrations up to 2 mM.

The search for additional inhibitors led to the identification of some BNIP derivatives as stronger inhibitors of TcSir2rp1. This class of compounds was previously described to be active at 10 μM, with few exceptions, against LiSir2rp1 [74]. However, no correlation has been established between the most active compounds for *L*. *infantum* and *T*. *cruzi* enzymes, suggesting that both enzymes might be targeted differentially by some of the derivatives [46]. In contrast to LiSir2rp1 inhibition, no structure-activity relationship regarding the length of the alkyl chain linking the two naphthalimidopropyl groups was observed for TcSir2rp1 [75]. Although a linker of eight carbons had no activity at 10 μM (compound **5**), when some of the carbons are substituted by heteroatoms like nitrogen (compound **9**) and oxygen (compound **9a**), the activity towards TcSir2rp1 increased significantly, suggesting that the rigidity of certain conformations and/or the ability to establish additional molecular interactions in this part of the molecule, increase inhibitory activity.

More importantly, some enzymatic inhibitors also demonstrated a strong anti-parasitic activity against *T*. *cruzi* epimastigotes, which for some was shown to be on-target, due to the increase in EC_50_ for the overexpressing cell-line. Moreover, amastigotes, the clinically relevant form responsible for infection persistence in humans, were shown to be sensitive to some of the BNIP derivatives. Differences observed between the enzymatic inhibition and the activity in the parasite may be due to distinct interactions between BNIPs and TcSir2rp1, and/or, possibly, by the contribution of a multi-target mechanism as well as the relative efficacy of entry into the host cell and then into the parasitophorous vacuole.

The majority of the compounds demonstrated low selectivity for the parasite and toxicity for host cells, as indicated by their selectivity indexes. A possible explanation is the characteristic DNA intercalation of BNIP derivatives that were originally developed as anti-cancer agents [86, 87]. While this might explain some of the toxic effects observed for host cells, it should not be excluded that DNA intercalation may be contributing to their reported anti-parasitic activity, especially since trypanosomes are susceptible to intercalating agents [88]. Analysis of TcSir2rp1 by Wregex and cNLS Mapper, bio-computational tools that identify nuclear export signals (NES) and nuclear localization signals (NLS), respectively, indicate the presence of non-canonical NES/NLS in the sequence of this sirtuin [89, 90]. Whether TcSir2rp1 does shuttle to and from the nucleus during specific times of the *T*. *cruzi* cell cycle, possibly to perform DNA damage repair like the *T*. *brucei* orthologue, remains to be reported. [42], In fact, if TcSir2rp1 also has a role in DNA repair both sirtuin inhibition and DNA intercalation may be synergistically contributing to the anti-parasitic activity. Activity towards other molecular targets should also not be excluded, especially considering that *T*. *cruzi* has another sirtuin, TcSir2rp3, that has also been described as having important functions in different cellular processes like metacyclogenesis, epimastigote growth and host-cell infectivity and replication [44, 45].

The docking studies also suggests that compound **9** is unable to distinguish between TcSir2rp1 and human SIRT2 proteins and is able to bind in multiple conformations either in the presence or absence of p53 substrate. This is likely due to the intrinsic hydrophobicity of sirtuin proteins and the hydrophobic nature of the two bisnapthalmidopropyl-moieties and the highly flexible linker between them in the BNIP derivatives.

Since BNIPSpd (**9**) met some of the biological criteria recommended for *T*. *cruzi* drug discovery follow-up, it was decided to further evaluate it as a possible lead compound [91]. The toxicity determination showed that although it has low SI values (around 1 for hepatocytes and neurons), the toxicity values in the μM range could be considered a promising starting point for further optimization. Moreover, the cardiotoxicity evaluation showed that cardiomyocyte function does not seem to be compromised. On the other hand, a more in-depth analysis of the toxicity by HCS showed that there is very limited injury to hepatocytes and neurons. BNIPSpd (**9**) showed no hepatotoxicity up to 12.5 μM. At 50 μM there is a low injury score mainly due to caspase 3/7 activation (although the viability -WST8- is also altered). In regard to neurotoxicity, as expected, the neurons start to suffer toxicity at lower concentrations; neurons showed no effects up to 0.7 μM but the injury score starts to rise in a dose dependent manner up to 12.5 μM. At higher concentrations (50 μM) the injury score is maintained at high levels. The mechanism of toxicity in neurons and hepatocytes seems to be the same, as the main parameter was caspase 3/7 activation followed by membrane integrity. At higher concentrations, all the parameters tested were affected. More assays need to be performed to evaluate the capacity of BNIPSpd (**9**) to cross the blood-brain barrier. In addition, BNIPSpd (**9**) showed decent aqueous solubility (up to 70%) and gastric stability (100%).

Assessment of efficacy of BNIPSpd (**9**) in mice infected with bioluminescent *T*. *cruzi* parasites by live imaging revealed lack of *in vivo* activity. The results may be explained by the pharmacokinetic profile of the compound, whose concentration in the blood always remained below the *in vitro* EC_50_ for amastigotes. The compound was no longer detected 3 hours after administration, even though the drug is administered by intravenous injection, the most bioavailable route. An explanation is that most of the compound is rapidly metabolized in the organism to an inactive form or removed from circulation. Medicinal chemistry modifications of the compound, as well as alternative formulations may improve activity and pharmacokinetics in the host organism.

## Supporting information

S1 Supporting Information (DOCX)

S1 Fig Schematic representation of the synthesis of compounds. cis BNIP-1,4-Dacyhex (**1a**), trans BNIP-1,4-Dacyhex (**1b**) and BNIP-1,4-Babenz (**1c**).
(TIF)

S2 Fig Validation of the epimastigote *T*. *cruzi* TcSir2rp1-modified cell lines by colony PCR. PCR validation of the genetically manipulated cell-lines: Lane 1: WT; Lane 2: over-expressing cell-line pTcINDEX TcSir2rp1: Hyg; Lane 3: over-expressing and single knockout cell-line TcSir2rp1ΔTcSir2rp::Pac pTcINDEX TcSir2rp1: Hyg; Lane 4: over-expressing and double knockout cell-line TcSir2rp1ΔTcSir2rp::Pac, Bsd pTcINDEX TcSir2rp1: Hyg. Primers used for ORF only are the TcSir2rp1 ORF forward and reverse primers, expected PCR product 1092bp. Primers used for the 5’UTR+ORF are the 5’ UTR F1 and TcSir2rp1 reverse ORF, expected size 1683bp.
Forward and reverse primers used for hygromycin resistance cassette (HYG), expected size 1041bp. Forward and reverse primers used for puromycin resistance cassette (PAC), expected size 610bp. Forward and reverse primers used for blasticidin resistance cassette (BSD), expected size 432bp. Forward and reverse ORF primers (5’-TATGCGGCCGCATGAATCAAGATAACGCCAAC-3’ and 5’- TATGGATCCTTATTTTCGGTCTGTCTGTGTGTACATG-3’ respectively. 5’ and 3’ UTR Primers respectively (F1: 5’-TATGCGGCCGCAGGAACCCACCACTTC-3’; R1: 5’- *CGTTTAAACTTACGGACCGTC*AAGCTTTGGGAAGAAGTAATCCACCT-3’; F2: 5’-*GACGGTCCGTAAGTTTAAACG*GGATCCACCGAAAATAAGAGGA-3’; R2: 5’-TATGCGGCCGCGATGCTCTTCATATTTATCTTGC-3’.
(TIF)

S3 Fig Development of the high-content screening assay against intracellular amastigotes of *Trypanosoma cruzi*. **A)** Representative images of the control conditions used in the assay: infected cells, non-infected cells and infected cells treated with 100 μM of benznidazole (BZN). **B)** Statistical validation of the screening assay based on the readout of parasites per cell, with a Z value of 0.68. **C)** Dose-response curve against the reference drug benznidazole, demonstrating and EC_50_ in line with previously published values [62].
(TIF)

S4 Fig Setting the *T*. *cruzi* infection in mice. **A)** *In vitro* detection limit of Luc^+^ trypomastigotes in a 96-well plate incubated with luciferin and imaged using the IVIS LUMINA LT. Circles and vertical lines represent the average ± standard deviation of the average radiance of quadruplicates. Statistical analysis was performed by standard t-test relative to luciferin background with no parasites. Significance is shown in asterisks (*, p-value ≤ 0.05). **B)** Comparison of different inocula of Luc^+^ trypomastigotes (10^4^, 10^5^, 10^6^) tested in BALB/c mice infections by quantification of the signal over 5 minutes (average radiance, photons/sec/cm^2^/sr) **C)** Representative images of the anesthetized bio-imaged mice infected with 10^4^ trypomastigotes at the time points before and after treatment (day 7 and 11 after infection, respectively).
(TIF)

S5 Fig *In vivo* efficacy testing of BNIPSpd (9). **A)** Mice were infected with 10^4^ Luc^+^ trypomastigotes by intraperitoneal injection. Treatments (BZN, benznidazole at 100 mg/kg/day *per os*, and BNIPSpd (**9**) at 5 mg/kg/day by intravenous injection) were initiated 7 days after infection, as well as the respective controls (KP, Kolliphor HS 15 20% *per os* and DMSO 10% by intravenous injection). Imaging was performed before treatment, at 7 days post-infection and after treatment, at 11 days post-infection, using an IVIS LUMINA LT and upon injection of 2.1 mg luciferin. In the lower panel, bioluminescence signal of whole mice represented in average radiance (photons/sec/cm^2^/sr) quantified before and after treatment. Data representative of two independent experiments. **B)** Snapshot Pharmacokinetics of BNIPSpd (**9**) in BALB/c mice by quantification of BNIPSpd (**9**) in the blood of mice by UHPLC-MS/MS ESI+ at different time-points after administration of a 5 mg/kg dose by intravenous injection. Data is the average of two independent experiments. The dashed line represents the value of EC_50_ for BNIPSpd (**9**) in the *in vitro* assay against *T*. *cruzi* amastigotes, and the dotted line represents the lower limit of quantification of the technique.
(TIF)

S6 Fig Superimposed structures of TcSir2rp1 and Human SIRT2. TcSir2rp1 structure in green, with p53 peptide carbons in pale blue. Human SIRT2 (4rmj) structure in purple, with carbons of ligands ADP and nicotinamide in pale pink.
(TIFF)

S7 Fig Docking of Human SIRT2 (apo structure 3zgo) with compound 9. A) image shows the same orientation as TcSir2rp1 with compound 9 (Fig 6B and 6C), B) is the same model rotated by 90° to view putative ligand binding pocket, with a zoomed in image on compound **9**. C) images showing an alternative mode of compound **9** binding.All of these have similar binding affinities to those observed for TcSir2rp1.
(TIFF)

S8 Fig Docking of Human SIRT2 (4rmj after removing ligands) with compound 9. A) image shows the same orientation of TcSir2rp1 with compound **9** (Fig 6B and 6C), B) is same model rotated 90°. C) is the same model turned by an additional 90°. D) is the same rotation as C, but with an alternative binding mode by compound 9, which is zoomed in on compound **9** (E). All of these have similar binding affinities to those observed for TcSir2rp1.
(TIFF)

S9 Fig TcSir2rp1 structure and docking with compound 1a. A) image shows the same orientation of TcSir2rp1 with compound **9** (Fig 6B and 6C), but with 1a docked, which is zoomed in on (B), and with an alternative binding orientation (C). D) Shows TcSir2rp1 in the presence of p53 and docked compound **1a**, which is zoomed in on (E), and with an alternative binding orientation (F). These all have very similar binding affinities 8–11 kcal/mol.
(TIFF)

S10 Fig TcSir2rp1 structure and docking with compound 1b. A) image shows the same orientation of TcSir2rp1 with compound 9 (Fig 6B and 6C), but with 1b docked, which is zoomed in on (B), and with an alternative binding orientation (C). D) Shows TcSir2rp1 in the presence of p53 and docked compound **1a**, which is zoomed in on (E), and with an alternative binding orientation (F). These all have very similar binding affinities 8–11 kcal/mol.
(TIFF)

## References

[1] WHO Chagas disease in Latin America: an epidemiological update based on 2010 estimates. Wkly Epidemiol Rec, 2015, 90(6), 33–43. 25671846

[2] Mathers C. D.; Ezzati M.; Lopez A. D. Measuring the burden of neglected tropical diseases: the global burden of disease framework. PLoS Negl Trop Dis, 2007, 1(2), e114 doi: 10.1371/journal.pntd.0000114 18060077 10.1371/journal.pntd.0000114 PMC2100367

[3] Lee B. Y.; Bacon K. M.; Bottazzi M. E.; Hotez P. J. Global economic burden of Chagas disease: a computational simulation model. Lancet Infect Dis, 2013, 13(4), 342–348. doi: 10.1016/S1473-3099(13)70002-1 23395248 10.1016/S1473-3099(13)70002-1 PMC3763184

[4] Garcia MN, Woc-Colburn L, Aguilar D, Hotez PJ, Murray KO (2015) Historical Perspectives on the Epidemiology of Human Chagas Disease in Texas and Recommendations for Enhanced Understanding of Clinical Chagas Disease in the Southern United States. PLoS Negl Trop Dis 9(11): e0003981 doi: 10.1371/journal.pntd.0003981 26540273 10.1371/journal.pntd.0003981 PMC4634991

[5] Bern C. Chagas' Disease. N Engl J Med, 2015, 373(5), 456–466. doi: 10.1056/NEJMra1410150 26222561 10.1056/NEJMra1410150

[6] Rassi A.; Marin-Neto J. Chagas disease. The Lancet, 2010, 375(9723), 1388–1402. 10.1016/S0140-6736(10)60061-X 20399979

[7] WHO Reporte sobre la enfermedade de Chagas. Available at: http://whqlibdoc.who.int/hq/2007/TDR_SWG_09_spa.pdf (Accessed May 22, 2015).

[8] Schmunis G. A.; Yadon Z. E. Chagas disease: a Latin American health problem becoming a world health problem. Acta Trop, 2010, 115(1–2), 14–21. doi: 10.1016/j.actatropica.2009.11.003 19932071 10.1016/j.actatropica.2009.11.003

[9] Viotti R.; Vigliano C.; Lococo B.; Alvarez M. G.; Petti M.; Bertocchi G.; Armenti A. Side effects of benznidazole as treatment in chronic Chagas disease: fears and realities. Expert Rev Anti Infect Ther, 2009, 7(2), 157–163. doi: 10.1586/14787210.7.2.157 19254164 10.1586/14787210.7.2.157

[10] Castro J. A.; de Mecca M. M.; Bartel L. C. Toxic side effects of drugs used to treat Chagas' disease (American trypanosomiasis). Hum Exp Toxicol, 2006, 25(8), 471–479. doi: 10.1191/0960327106het653oa 16937919 10.1191/0960327106het653oa

[11] Clayton J. Chagas disease: pushing through the pipeline. Nature, 2010, 465(7301), S12–S15. doi: 10.1038/nature09224 20571548 10.1038/nature09224

[12] Molina I.; Gomez i Prat J.; Salvador F.; Trevino B.; Sulleiro E.; Serre N.; Pou D.; Roure S.; Cabezos J.; Valerio L.; Blanco-Grau A.; Sanchez-Montalva A.; Vidal X.; Pahissa A. Randomized trial of posaconazole and benznidazole for chronic Chagas' disease. N Engl J Med, 2014, 370(20), 1899–1908. doi: 10.1056/NEJMoa1313122 24827034 10.1056/NEJMoa1313122

[13] Morillo C. A.; Marin-Neto J. A.; Avezum A.; Sosa-Estani S.; Rassi A. Jr.; Rosas F.; Villena E.; Quiroz R.; Bonilla R.; Britto C.; Guhl F.; Velazquez E.; Bonilla L.; Meeks B.; Rao-Melacini P.; Pogue J.; Mattos A.; Lazdins J.; Rassi A.; Connolly S. J.; Yusuf S.; Investigators B. Randomized Trial of Benznidazole for Chronic Chagas' Cardiomyopathy. N Engl J Med, 2015, 373(14), 1295–1306. doi: 10.1056/NEJMoa1507574 26323937 10.1056/NEJMoa1507574

[14] DNDi, Drug Trial for Leading Parasitic Killer of the Americas Shows Mixed Results but Provides New Evidence for Improved Therapy. Washington D.C., 2013.

[15] Frye R. A. Phylogenetic classification of prokaryotic and eukaryotic Sir2-like proteins. Biochem Biophys Res Commun, 2000, 273(2), 793–798. doi: 10.1006/bbrc.2000.3000 10873683 10.1006/bbrc.2000.3000

[16] Costantini S.; Sharma A.; Raucci R.; Costantini M.; Autiero I.; Colonna G. Genealogy of an ancient protein family: the Sirtuins, a family of disordered members. BMC Evol Biol, 2013, 13, 60 doi: 10.1186/1471-2148-13-60 23497088 10.1186/1471-2148-13-60 PMC3599600

[17] Denu J. M. Linking chromatin function with metabolic networks: Sir2 family of NAD(+)-dependent deacetylases. Trends Biochem Sci, 2003, 28(1), 41–48. 12517451 10.1016/s0968-0004(02)00005-1

[18] Liou G. G.; Tanny J. C.; Kruger R. G.; Walz T.; Moazed D. Assembly of the SIR complex and its regulation by O-acetyl-ADP-ribose, a product of NAD-dependent histone deacetylation. Cell, 2005, 121(4), 515–527. doi: 10.1016/j.cell.2005.03.035 15907466 10.1016/j.cell.2005.03.035

[19] Haigis M. C.; Mostoslavsky R.; Haigis K. M.; Fahie K.; Christodoulou D. C.; Murphy A. J.; Valenzuela D. M.; Yancopoulos G. D.; Karow M.; Blander G.; Wolberger C.; Prolla T. A.; Weindruch R.; Alt F. W.; Guarente L. SIRT4 inhibits glutamate dehydrogenase and opposes the effects of calorie restriction in pancreatic beta cells. Cell, 2006, 126(5), 941–954. doi: 10.1016/j.cell.2006.06.057 16959573 10.1016/j.cell.2006.06.057

[20] Liszt G.; Ford E.; Kurtev M.; Guarente L. Mouse Sir2 homolog SIRT6 is a nuclear ADP-ribosyltransferase. J Biol Chem, 2005, 280(22), 21313–21320. doi: 10.1074/jbc.M413296200 15795229 10.1074/jbc.M413296200

[21] Garcia-Salcedo J. A.; Gijon P.; Nolan D. P.; Tebabi P.; Pays E. A chromosomal SIR2 homologue with both histone NAD-dependent ADP-ribosyltransferase and deacetylase activities is involved in DNA repair in Trypanosoma brucei. EMBO J, 2003, 22(21), 5851–5862. doi: 10.1093/emboj/cdg553 14592982 10.1093/emboj/cdg553 PMC275410

[22] Tavares J.; Ouaissi A.; Santarem N.; Sereno D.; Vergnes B.; Sampaio P.; Cordeiro-da-Silva A. The Leishmania infantum cytosolic SIR2-related protein 1 (LiSIR2RP1) is an NAD+ -dependent deacetylase and ADP-ribosyltransferase. Biochem J, 2008, 415(3), 377–386. doi: 10.1042/BJ20080666 18598238 10.1042/BJ20080666

[23] Tanner K. G.; Landry J.; Sternglanz R.; Denu J. M. Silent information regulator 2 family of NAD- dependent histone/protein deacetylases generates a unique product, 1-O-acetyl-ADP-ribose. Proc Natl Acad Sci U S A, 2000, 97(26), 14178–14182. doi: 10.1073/pnas.250422697 11106374 10.1073/pnas.250422697 PMC18891

[24] Du J.; Zhou Y.; Su X.; Yu J. J.; Khan S.; Jiang H.; Kim J.; Woo J.; Kim J. H.; Choi B. H.; He B.; Chen W.; Zhang S.; Cerione R. A.; Auwerx J.; Hao Q.; Lin H. Sirt5 is a NAD-dependent protein lysine demalonylase and desuccinylase. Science, 2011, 334(6057), 806–809. doi: 10.1126/science.1207861 22076378 10.1126/science.1207861 PMC3217313

[25] Tan M.; Peng C.; Anderson K. A.; Chhoy P.; Xie Z.; Dai L.; Park J.; Chen Y.; Huang H.; Zhang Y.; Ro J.; Wagner G. R.; Green M. F.; Madsen A. S.; Schmiesing J.; Peterson B. S.; Xu G.; Ilkayeva O. R.; Muehlbauer M. J.; Braulke T.; Muhlhausen C.; Backos D. S.; Olsen C. A.; McGuire P. J.; Pletcher S. D.; Lombard D. B.; Hirschey M. D.; Zhao Y. Lysine glutarylation is a protein posttranslational modification regulated by SIRT5. Cell Metab, 2014, 19(4), 605–617. doi: 10.1016/j.cmet.2014.03.014 24703693 10.1016/j.cmet.2014.03.014 PMC4108075

[26] Jeong S. M.; Haigis M. C. Sirtuins in Cancer: a Balancing Act between Genome Stability and Metabolism. Mol Cells, 2015, 38(9), 750–758. doi: 10.14348/molcells.2015.0167 26420294 10.14348/molcells.2015.0167 PMC4588717

[27] Gomes P.; Fleming Outeiro T.; Cavadas C. Emerging Role of Sirtuin 2 in the Regulation of Mammalian Metabolism. Trends Pharmacol Sci, 2015. 10.1016/j.tips.2015.08.001 26538315

[28] Watroba M.; Szukiewicz D. The role of sirtuins in aging and age-related diseases. Adv Med Sci, 2015, 61(1), 52–62. doi: 10.1016/j.advms.2015.09.003 26521204 10.1016/j.advms.2015.09.003

[29] Mostoslavsky R.; Esteller M.; Vaquero A. At the crossroad of lifespan, calorie restriction, chromatin and disease: meeting on sirtuins. Cell Cycle, 2010, 9(10), 1907–1912. doi: 10.4161/cc.9.10.11481 20458180 10.4161/cc.9.10.11481

[30] Seto E.; Yoshida M. Erasers of histone acetylation: the histone deacetylase enzymes. Cold Spring Harb Perspect Biol, 2014, 6(4), a018713 doi: 10.1101/cshperspect.a018713 24691964 10.1101/cshperspect.a018713 PMC3970420

[31] Michishita E.; Park J. Y.; Burneskis J. M.; Barrett J. C.; Horikawa I. Evolutionarily conserved and nonconserved cellular localizations and functions of human SIRT proteins. Mol Biol Cell, 2005, 16(10), 4623–4635. doi: 10.1091/mbc.E05-01-0033 16079181 10.1091/mbc.E05-01-0033 PMC1237069

[32] Muth V.; Nadaud S.; Grummt I.; Voit R. Acetylation of TAF(I)68, a subunit of TIF-IB/SL1, activates RNA polymerase I transcription. EMBO J, 2001, 20(6), 1353–1362. doi: 10.1093/emboj/20.6.1353 11250901 10.1093/emboj/20.6.1353 PMC145524

[33] Mao Z.; Tian X.; Van Meter M.; Ke Z.; Gorbunova V.; Seluanov A. Sirtuin 6 (SIRT6) rescues the decline of homologous recombination repair during replicative senescence. Proc Natl Acad Sci U S A, 2012, 109(29), 11800–11805. doi: 10.1073/pnas.1200583109 22753495 10.1073/pnas.1200583109 PMC3406824

[34] Mao Z.; Hine C.; Tian X.; Van Meter M.; Au M.; Vaidya A.; Seluanov A.; Gorbunova V. SIRT6 promotes DNA repair under stress by activating PARP1. Science, 2011, 332(6036), 1443–1446. doi: 10.1126/science.1202723 21680843 10.1126/science.1202723 PMC5472447

[35] Karim M. F.; Yoshizawa T.; Sato Y.; Sawa T.; Tomizawa K.; Akaike T.; Yamagata K. Inhibition of H3K18 deacetylation of Sirt7 by Myb-binding protein 1a (Mybbp1a). Biochem Biophys Res Commun, 2013, 441(1), 157–163. doi: 10.1016/j.bbrc.2013.10.020 24134843 10.1016/j.bbrc.2013.10.020

[36] Tsai Y. C.; Greco T. M.; Boonmee A.; Miteva Y.; Cristea I. M. Functional proteomics establishes the interaction of SIRT7 with chromatin remodeling complexes and expands its role in regulation of RNA polymerase I transcription. Mol Cell Proteomics, 2012, 11(5), 60–76. doi: 10.1074/mcp.A111.015156 22586326 10.1074/mcp.A111.015156 PMC3418843

[37] North B. J.; Verdin E. Interphase nucleo-cytoplasmic shuttling and localization of SIRT2 during mitosis. PLoS One, 2007, 2(8), e784 doi: 10.1371/journal.pone.0000784 17726514 10.1371/journal.pone.0000784 PMC1949146

[38] Hirschey M. D.; Shimazu T.; Goetzman E.; Jing E.; Schwer B.; Lombard D. B.; Grueter C. A.; Harris C.; Biddinger S.; Ilkayeva O. R.; Stevens R. D.; Li Y.; Saha A. K.; Ruderman N. B.; Bain J. R.; Newgard C. B.; Farese R. V. Jr.; Alt F. W.; Kahn C. R.; Verdin E. SIRT3 regulates mitochondrial fatty-acid oxidation by reversible enzyme deacetylation. Nature, 2010, 464(7285), 121–125. doi: 10.1038/nature08778 20203611 10.1038/nature08778 PMC2841477

[39] Nakagawa T.; Lomb D. J.; Haigis M. C.; Guarente L. SIRT5 Deacetylates carbamoyl phosphate synthetase 1 and regulates the urea cycle. Cell, 2009, 137(3), 560–570. doi: 10.1016/j.cell.2009.02.026 19410549 10.1016/j.cell.2009.02.026 PMC2698666

[40] Freitas-Junior L. H.; Hernandez-Rivas R.; Ralph S. A.; Montiel-Condado D.; Ruvalcaba-Salazar O. K.; Rojas-Meza A. P.; Mancio-Silva L.; Leal-Silvestre R. J.; Gontijo A. M.; Shorte S.; Scherf A. Telomeric heterochromatin propagation and histone acetylation control mutually exclusive expression of antigenic variation genes in malaria parasites. Cell, 2005, 121(1), 25–36. doi: 10.1016/j.cell.2005.01.037 15820676 10.1016/j.cell.2005.01.037

[41] Tonkin C. J.; Carret C. K.; Duraisingh M. T.; Voss T. S.; Ralph S. A.; Hommel M.; Duffy M. F.; Silva L. M.; Scherf A.; Ivens A.; Speed T. P.; Beeson J. G.; Cowman A. F. Sir2 paralogues cooperate to regulate virulence genes and antigenic variation in Plasmodium falciparum. PLoS Biol, 2009, 7(4), e84 doi: 10.1371/journal.pbio.1000084 19402747 10.1371/journal.pbio.1000084 PMC2672602

[42] Alsford S.; Kawahara T.; Isamah C.; Horn D. A sirtuin in the African trypanosome is involved in both DNA repair and telomeric gene silencing but is not required for antigenic variation. Mol Microbiol, 2007, 63(3), 724–736. doi: 10.1111/j.1365-2958.2006.05553.x 17214740 10.1111/j.1365-2958.2006.05553.x

[43] Vergnes B.; Sereno D.; Tavares J.; Cordeiro-da-Silva A.; Vanhille L.; Madjidian-Sereno N.; Depoix D.; Monte-Alegre A.; Ouaissi A. Targeted disruption of cytosolic SIR2 deacetylase discloses its essential role in Leishmania survival and proliferation. Gene, 2005, 363, 85–96. doi: 10.1016/j.gene.2005.06.047 16236469 10.1016/j.gene.2005.06.047

[44] Moretti N. S.; Augusto L. D.; Clemente T. M.; Antunes R. P.; Yoshida N.; Torrecilhas A. C.; Cano M. I.; Schenkman S. Characterization of Trypanosoma cruzi sirtuins as possible drug targets for Chagas Disease. Antimicrob Agents Chemother, 2015, 59(8), 4669–79. doi: 10.1128/AAC.04694-14 26014945 10.1128/AAC.04694-14 PMC4505258

[45] Ritagliati C.; Alonso V. L.; Manarin R.; Cribb P.; Serra E. C. Overexpression of cytoplasmic TcSIR2RP1 and mitochondrial TcSIR2RP3 impacts on Trypanosoma cruzi growth and cell invasion. PLoS Negl Trop Dis, 2015, 9(4), e0003725 doi: 10.1371/journal.pntd.0003725 25875650 10.1371/journal.pntd.0003725 PMC4398437

[46] Tavares J.; Ouaissi A.; Kong Thoo Lin P.; Loureiro I.; Kaur S.; Roy N.; Cordeiro-da-Silva A. Bisnaphthalimidopropyl derivatives as inhibitors of Leishmania SIR2 related protein 1. ChemMedChem, 2010, 5(1), 140–147. doi: 10.1002/cmdc.200900367 19937668 10.1002/cmdc.200900367

[47] Andriani G.; Chessler A. D.; Courtemanche G.; Burleigh B. A.; Rodriguez A. Activity in vivo of anti-Trypanosoma cruzi compounds selected from a high throughput screening. PLoS Negl Trop Dis, 2011, 5(8), e1298 doi: 10.1371/journal.pntd.0001298 21912715 10.1371/journal.pntd.0001298 PMC3166044

[48] Vazquez M. P.; Levin M. J. Functional analysis of the intergenic regions of TcP2beta gene loci allowed the construction of an improved Trypanosoma cruzi expression vector. Gene, 1999, 239(2), 217–225. 10548722 10.1016/s0378-1119(99)00386-8

[49] Taylor M. C. & Kelly J. M. pTcINDEX: a stable tetracycline-regulated expression vector for Trypanosoma cruzi BMC biotechnology, 6, 32 doi: 10.1186/1472-6750-6-32 16824206 10.1186/1472-6750-6-32 PMC1544328

[50] Graça NAG, Gaspar L, Costa DM, Loureiro I, Thoo-Lin PK, Ramos I, Roura M, Pruvost A, Pemberton IK, Loukil H, Macdougall J, Tavares J, Cordeiro-da-Silva A. 2016 Activity of bisnaphthalimidopropyl derivatives against *Trypanosoma brucei*. Antimicrob Agents Chemother 60:2532–2536. doi: 10.1128/AAC.02490-15 26787703 10.1128/AAC.02490-15 PMC4808195

[51] Lin P. K.; Pavlov V. A. The synthesis and in vitro cytotoxic studies of novel bis-naphthalimidopropyl polyamine derivatives. Bioorg Med Chem Lett, 2000, 10(14), 1609–1612. 10915063 10.1016/s0960-894x(00)00293-6

[52] Barron G. A.; Bermano G.; Gordon A.; Kong Thoo Lin P. Synthesis, cytotoxicity and DNA-binding of novel bisnaphthalimidopropyl derivatives in breast cancer MDA-MB-231 cells. Eur J Med Chem, 2010, 45(4), 1430–1437. doi: 10.1016/j.ejmech.2009.12.047 20064676 10.1016/j.ejmech.2009.12.047

[53] Lima T., Barron R.; A., Grabowska G.; A., Bermano J., Kaur G., Roy S., Helena Vasconcelos N., K.T M. Lin P. Cytotoxicity and Cell Death Mechanisms Induced by a Novel Bisnaphthalimidopropyl Derivative against the NCI-H460 non-small Lung Cancer Cell Line. Anti-Cancer Agents in Medicinal Chemistry, 2013, 13(3), 414–421. 23092269 10.2174/1871520611313030005

[54] Kabsch W. XDS. Acta Crystallographica. Section D Biological Crystallography. 2010; 66(Pt 2): 25–132 10.1107/S0907444909047337 PMC2815665 20124692

[55] Winn M.D.; Ballard C.C.; Cowtan K.D.; Dodson E.J.; Emsley P.; Evans P.R.; Keegan R.M.; Krissinel E.B.; Leslie A.G.; McCoy A.; McNicholas S.J.; Murshudov G.N.; Pannu N.S.; Potterton E.A.; Powell H.R.; Read R.J.; Vagin A.; Wilson K.S. Overview of the CCP4 suite and current developments. Acta Crystallographica. Section D Biological Crystallography. 2011; 67 (Pt 4):235–242 21460441 10.1107/S0907444910045749 PMC3069738

[56] Keegan R.M.; Winn M.D. Automated search-model discovery and preparation for structure solution by molecular replacement. Acta Crystallographica. Section D Biological Crystallography. 2007; 63 (Pt 4):447–457. 17372348 10.1107/S0907444907002661

[57] Emsley P.; Lohkamp B.; Scott W. G.; Cowtan K. Features and development of Coot. Acta Crystallographica. Section D Biological Crystallography. 2010; 66 (Pt 4), 486–501 20383002 10.1107/S0907444910007493 PMC2852313

[58] Murshudov G.N.; Skubak P.; Lebedev A.A.; Pannu N.S.; Steiner R.A.; Nicholls R.A.; Winn M.D.; Long F.; Vagin A.A. REFMAC5 for the refinement of macromolecular crystal structures. Acta Crystallographica. Section D Biological Crystallography. 2011 67 (Pt 4): 355–367. 21460454 10.1107/S0907444911001314 PMC3069751

[59] Berman H. M., Westbrook J., Feng Z., Gilliland G., Bhat T. N., Weissig H., Shindyalov I. N., Bourne P. E. (2000) The Protein Data Bank. *Nucleic Acids Res*, 28 (1): 235–242. 10592235 10.1093/nar/28.1.235 PMC102472

[60] Zhao K.; Harshaw R.; Chai X.; Marmorstein R. Structural basis for nicotinamide cleavage and ADP-ribose transfer by NAD(+)-dependent Sir2 histone/protein deacetylases. Proc Natl Acad Sci U S A. 2004 101(23):8563–8. doi: 10.1073/pnas.0401057101 15150415 10.1073/pnas.0401057101 PMC423234

[61] Moniot S, Schutkowski M, Steegborn C. (2013) Crystal structure analysis of human Sirt2 and its ADP-ribose complex. *J Struct Biol*.,182(2):136–43 doi: 10.1016/j.jsb.2013.02.012 23454361 10.1016/j.jsb.2013.02.012

[62] Rumpf T., Schiedel M., Karaman B., Roessler C., North B. J., Lehotzky A., Oláh J., Ladwein K. I., Schmidtkunz K., Gajer M., Pannek M., Steegborn C., Sinclair D. A., Gerhardt S., Ovádi J., Schutkowski M., Sippl W., Einsle O., Jung M. (2015) Selective Sirt2 inhibition by ligand-induced rearrangement of the active site. *Nat Commun*., 6:6263 doi: 10.1038/ncomms7263 25672491 10.1038/ncomms7263 PMC4339887

[63] Morris G. M., Huey R., Lindstrom W., Sanner M. F., Belew R. K., Goodsell D. S. and Olson A. J. (2009) Autodock4 and AutoDockTools4: automated docking with selective receptor flexiblity. *J*. *Computational Chemistry*, 16: 2785–91 10.1002/jcc.21256 PMC2760638 19399780

[64] Trott O., and Olson A. J. (2010) AutoDock Vina: improving the speed and accuracy of docking with a new scoring function, efficient optimization and multithreading, *J*. *Computational Chemistry*, 31: 455–461 10.1002/jcc.21334 PMC3041641 19499576

[65] Schrödinger, The PyMOL Molecular Graphics System, Version 1.5.0.4 Schrödinger, LLC.

[66] van Meerloo J.; Kaspers G. L.; Cloos J. Cell Sensitivity Assays: The MTT Assay In: Cancer Cell Culture; Cree I. A., Ed. Humana Press: 2011; Vol. 731, pp. 237–245. 10.1007/978-1-61779-080-5_20 21516412

[67] Kronenwerth M., Dauth C., Kaiser M., Pemberton I. and Bode H. B. (2014), Facile Synthesis of Cyclohexanediones and Dialkylresorcinols–Bioactive Natural Products from Entomopathogenic Bacteria. Eur. J. Org. Chem., 2014: 8026–8028.

[68] Kowieski T. M.; Lee S.; Denu J. M. Acetylation-dependent ADP-ribosylation by Trypanosoma brucei Sir2. J Biol Chem, 2008, 283(9), 5317–5326. doi: 10.1074/jbc.M707613200 18165239 10.1074/jbc.M707613200

[69] Borra M. T.; Langer M. R.; Slama J. T.; Denu J. M. Substrate specificity and kinetic mechanism of the Sir2 family of NAD+-dependent histone/protein deacetylases. Biochemistry, 2004, 43(30), 9877–9887. doi: 10.1021/bi049592e 15274642 10.1021/bi049592e

[70] North B. J.; Marshall B. L.; Borra M. T.; Denu J. M.; Verdin E. The Human Sir2 Ortholog, SIRT2, Is an NAD+-Dependent Tubulin Deacetylase. Molecular Cell, 2003, 11(2), 437–444. 12620231 10.1016/s1097-2765(03)00038-8

[71] Avalos J. L.; Bever K. M.; Wolberger C. Mechanism of sirtuin inhibition by nicotinamide: altering the NAD(+) cosubstrate specificity of a Sir2 enzyme. Mol Cell, 2005, 17(6), 855–868. doi: 10.1016/j.molcel.2005.02.022 15780941 10.1016/j.molcel.2005.02.022

[72] Chakrabarty S. P.; Saikumari Y. K.; Bopanna M. P.; Balaram H. Biochemical characterization of Plasmodium falciparum Sir2, a NAD+-dependent deacetylase. Mol Biochem Parasitol, 2008, 158(2), 139–151. doi: 10.1016/j.molbiopara.2007.12.003 18221799 10.1016/j.molbiopara.2007.12.003

[73] Soares M. B.; Silva C. V.; Bastos T. M.; Guimaraes E. T.; Figueira C. P.; Smirlis D.; Azevedo W. F. Jr. Anti-Trypanosoma cruzi activity of nicotinamide. Acta Trop, 2012, 122(2), 224–229. doi: 10.1016/j.actatropica.2012.01.001 22281243 10.1016/j.actatropica.2012.01.001

[74] Tavares J.; Ouaissi A.; Silva A. M.; Lin P. K.; Roy N.; Cordeiro-da-Silva A. Anti-leishmanial activity of the bisnaphthalimidopropyl derivatives. Parasitol Int, 2012, 61(2), 360–363. doi: 10.1016/j.parint.2011.11.005 22155672 10.1016/j.parint.2011.11.005

[75] Zhang J. H.; Chung T. D.; Oldenburg K. R. A Simple Statistical Parameter for Use in Evaluation and Validation of High Throughput Screening Assays. J Biomol Screen, 1999, 4(2), 67–73. doi: 10.1177/108705719900400206 10838414 10.1177/108705719900400206

[76] Moraes C. B.; Giardini M. A.; Kim H.; Franco C. H.; Araujo-Junior A. M.; Schenkman S.; Chatelain E.; Freitas-Junior L. H. Nitroheterocyclic compounds are more efficacious than CYP51 inhibitors against Trypanosoma cruzi: implications for Chagas disease drug discovery and development. Sci Rep, 2014, 4. 10.1038/srep04703 PMC4004771 24736467

[77] Mingatto F. E.; Rodrigues T.; Pigoso A. A.; Uyemura S. A.; Curti C.; Santos A. C. The critical role of mitochondrial energetic impairment in the toxicity of nimesulide to hepatocytes. J Pharmacol Exp Ther, 2002, 303(2), 601–607. doi: 10.1124/jpet.102.038620 12388641 10.1124/jpet.102.038620

[78] Tripathi R.; Tripathi P.; Pancholi S. S.; Patel C. N. The genotoxic and cytotoxic effects of nimesulide in the mouse bone marrow. Drug Chem Toxicol, 2014, 37(3), 255–260. doi: 10.3109/01480545.2013.838779 24164450 10.3109/01480545.2013.838779

[79] Borkotoky D.; Panda S. K.; Sahoo G. R.; Parija S. C. Genotoxicity of nimesulide in Wistar rats. Drug Chem Toxicol, 2014, 37(2), 178–183. doi: 10.3109/01480545.2013.834357 24116684 10.3109/01480545.2013.834357

[80] Religa A. A.; Waters A. P. Sirtuins of parasitic protozoa: in search of function(s). Mol Biochem Parasitol, 2012, 185(2), 71–88. doi: 10.1016/j.molbiopara.2012.08.003 22906508 10.1016/j.molbiopara.2012.08.003 PMC3484402

[81] Zheng W. Sirtuins as emerging anti-parasitic targets. Eur J Med Chem, 2013, 59, 132–140. doi: 10.1016/j.ejmech.2012.11.014 23220641 10.1016/j.ejmech.2012.11.014

[82] Li F.; Chong Z. Z.; Maiese K. Cell Life versus cell longevity: the mysteries surrounding the NAD+ precursor nicotinamide. Curr Med Chem, 2006, 13(8), 883–895. 16611073 10.2174/092986706776361058 PMC2248696

[83] Prusty D.; Mehra P.; Srivastava S.; Shivange A. V.; Gupta A.; Roy N.; Dhar S. K. Nicotinamide inhibits Plasmodium falciparum Sir2 activity in vitro and parasite growth. FEMS Microbiol Lett, 2008, 282(2), 266–272. doi: 10.1111/j.1574-6968.2008.01135.x 18397290 10.1111/j.1574-6968.2008.01135.x

[84] Sereno D.; Alegre A. M.; Silvestre R.; Vergnes B.; Ouaissi A. In vitro antileishmanial activity of nicotinamide. Antimicrob Agents Chemother, 2005, 49(2), 808–812. doi: 10.1128/AAC.49.2.808-812.2005 15673775 10.1128/AAC.49.2.808-812.2005 PMC547366

[85] Unciti-Broceta J. D.; Maceira J.; Morales S.; Garcia-Perez A.; Munoz-Torres M. E.; Garcia-Salcedo J. A. Nicotinamide inhibits the lysosomal cathepsin b-like protease and kills African trypanosomes. J Biol Chem, 2013, 288(15), 10548–10557. doi: 10.1074/jbc.M112.449207 23443665 10.1074/jbc.M112.449207 PMC3624436

[86] Ralton L.; Bestwick C. S.; Thoo Lin P. K. Polyamine Analogues and Derivatives as Potential Anticancer Agents. Current Bioactive Compounds, 2007, 3(3), 179–191.

[87] Braña M. F.; Castellano J. M.; Morán M.; Pérez de Vega M. J.; Qian X. D.; Romerdahl C. A.; Keilhauer G. Bis-naphthalimides. 2. Synthesis and biological activity of 5,6-acenaphthalimidoalkyl-1,8-naphthalimidoalkyl amines. European Journal of Medicinal Chemistry, 1995, 30(3), 235–239.

[88] Roy Chowdhury A.; Bakshi R.; Wang J.; Yildirir G.; Liu B.; Pappas-Brown V.; Tolun G.; Griffith J. D.; Shapiro T. A.; Jensen R. E.; Englund P. T. The killing of African trypanosomes by ethidium bromide. PLoS Pathog, 2010, 6(12), e1001226 doi: 10.1371/journal.ppat.1001226 21187912 10.1371/journal.ppat.1001226 PMC3002999

[89] Prieto G.; Fullaondo A.; Rodriguez J. A. Prediction of nuclear export signals using weighted regular expressions (Wregex). Bioinformatics, 2014, 30(9), 1220–1227. doi: 10.1093/bioinformatics/btu016 24413524 10.1093/bioinformatics/btu016

[90] Kosugi S.; Hasebe M.; Tomita M.; Yanagawa H. Systematic identification of cell cycle-dependent yeast nucleocytoplasmic shuttling proteins by prediction of composite motifs. Proc Natl Acad Sci U S A, 2009, 106(25), 10171–10176. doi: 10.1073/pnas.0900604106 19520826 10.1073/pnas.0900604106 PMC2695404

[91] Owens J. Determining druggability. Nat Rev Drug Discov, 2007, 6(3), 187–187.

