# Supplementary material for: Inhibitors of Trypanosoma cruzi Sir2 related protein 1 as potential drugs against Chagas disease
Source: PLoS Negl Trop Dis. 2018 Jan 22;12(1):e0006180. doi: 10.1371/journal.pntd.0006180 (PMC5794198; doi:10.1371/journal.pntd.0006180)
Supplement: S1 Supporting Information — (DOCX) [file pntd.0006180.s001.docx]

**Supporting information**

**Inhibitors of *Trypanosoma cruzi* Sir2 related protein 1 as potential drugs against Chagas disease**

**Synthesis of BNIP derivatives**

All starting materials, purchased from Sigma-Aldrich, were of research-grade quality and were used without further purification. Thin Layer Chromatography (TLC) was performed on silica gel 60 F254 aluminium plates (Merck) in dichloromethane/methanol (95:5). NMR was recorded on a Bruker 400 Ultra shield spectrometer at 400.1 MHz for ^1^H and 100.6 MHz for ^13^C.

***BNIP-1,4-Dacychex, 1a, trans BNIP-1,4-Dacyhex, 1b, and BNIP-1,4-Dabenz, 1c.*** All the above diamines were treated with mesityl-chloride in pyridine to yield the corresponding bismesityl derivatives in quantitative yields. Subsequent N-alkylation reaction with O-tosylpropylnaphthalimide gave the corresponding protected bisnaphthalimidopropyl derivatives, which upon deprotection with HBr/glacial acetic acid in dichloromethane [51] yielded **1a, 1b** and **1c** (Fig 1).

**Cis BNIP-1,4-Dacyhex, 1a.** HRMS (ESI): calculated for C_36_H_37_N_4_O_4_: 589.2809 ([M+H]^+^), found: 589.2798 ([M+H]^+^0.

**Trans BNIP-1,4-Dacyhex, 1b.** After the N-alkylation step, the crude synthesize was thoroughly dried in a vacuum oven. The pale brown solid was re-suspended in hot toluene. The latter was quickly filtered and washed with hot toluene. The resulting precipitate was dried in *vacuo* and the product obtained gave a single spot on TLC (64%) as the protected trans BNIP-1,4-Dacyhex. The dibromide salt was obtained in quantitative yield.

^1^H NMR (400.1 MHz, DMSO-d_6_): δ 8.70-7.60 (m, aromatic naphthalimido ring protons), 4.20 (t, 2 x CH_2_-N), 3.40(s, CH_2_-N), 3.10 (s, NH), 2.20-2.10 (m, CH_2_), 1.50 (t, CH). ^13^C NMR (DMSO): δ 164.26 (C=O), 134.92-122.64 (Aromatic protons from the Naphthlimido rings), 55.06 (CH), 42.74 (CH_2_), 37.59 (CH_2_), 26.91 (CH_2_).

HRMS (ESI): calculated for C_36_H_36_N_4_O_4_ 2HBr: 748.1261, 589.2809 ([M+H]^+^-2HBr), found: 589.2808 ([M+H]^+^-2HBr).

**BNIP-1,4-Dabenz, 1c.** *p*-phenylenediamine (1 g; 9.25 mmol) was dissolved in pyridine (20 mL) and the solution was stirred. Mesithylenesulphonyl-chloride (4.25 g; 19.425 mmol) (2.1 M excess) was then added and the reaction was left stirring for 4h at room temperature. After this time, pyridine was removed, the remaining liquid was poured in icy water (200 mL) and a precipitate was formed. The latter was filtered off and washed with water several times. The solid was dried in the vacuum oven overnight to afford N,N-bismesityldiaminobenzene in 93.3% yield.

^1^H NMR (DMSO-d_6_): δ 9.9 (2H, NH), 6.9 (4H, s, Aromatic H), 6.8 (4H, s, aromatic H), 2.4 (12H, s, 4 x CH_3_), 2.2 (6H, s, 2 x CH_3_). ^13^C NMR (DMSO-d_6_): δ 142.3-122.0 (aromatic carbons), 22.8 (CH_2_), 20.8 (CH_2_).

N, N-bismesityldiaminobenzene (0.1 g) was dissolved in DMF (2.5 mL), followed by the addition of O-tosylpropylnaphthalimide (2.4 M excess) immediately followed by cesium carbonate (5 M excess). The solution was left stirring overnight at 70°C. Reaction completion was monitored by thin layer chromatography. The solution was poured into icy water (80 mL) to form a precipitate. The precipitate was filtered, washed thoroughly with water and dried in *vacuo*. Fully protected N,N-bismesityldiaminobenzene: ^1^H NMR (400.1 MHz, CDCl_3_): δ 8.50-7.7o (m, aromatic protons), 7.30, 6.80 (d, mesityl aromatic protons), 7.20 (s, diaminobenzene aromatic protons), 4.2 (t, CH_2_-N), 3.8 (t, CH_2_-N), 2.4 (s, Ar-CH_3_), 2.2 (s, Ar-CH_3_), 1.9 ppm (t, CH_2_). ^13^C (100.6 MHz, CDCl_3_): δ 164.00 (C=O), 134.00-122.00 (aromatic carbons), 143.00-132.00 (Mesityl aromatic carbons), 140.00-138.00 (diaminobenzene aromatic carbons), 48.00, 38.00, 27.00 (3 x CH_2_), 23.00, 21.00 ppm (Mesityl-CH_3_).

Protected BNIP-1,4-Dabenz (0.1 g; 0.106 mmol) was dissolved in DCM (5 mL) with heating and stirring. HBr/CH_3_COOH solution (385 µL) was added and the solution was left in the fume cupboard at room temperature for four days to form very fine precipitate which was centrifuged, washed with DCM and ether and centrifuged after each wash to give the final product BNIP-1,4-Dabenz **1c** (23% yield). ^1^H (DMSO-d_6_): 8.51-8.40, 7.89-7.84, 7.19-6.90 (Aromatic protons from the naphthalimido and benzene rings, 4.15-4.10 (4H, 2xCH_2_), 3.22-3.19 (4H, 2xCH_2_), 1.99-1.92 (4H, 2xCH_2_). ^13^C NMR (DMSO-d_6_): δ 164.10 (C=O), 134.80-122.60 (aromatic carbons), 55.60 (CH_2_), 37.90 (CH_2_).

LRMS for C_36_H_30_N_4_O_4_ was 742.09, 582.23 [M-2HBr]^+^, found: 582.20 [M-2HBr]^+^, 583.20 ([M+H]^+^) and 605.0 m/z ([M+Na]^+^).

**BNIPDPP salt, 6a.** In a round bottom flask (50 cm^3^), 1,3-bis-(4-piperidyl)propane (0.25 g, 1.19 x 10^-3^ mol,) and toluenesulfonyloxypropylnaphthalamide (0.98 g, 2.39 x 10^-3^ mol) were dissolved in THF(6 mL). The reaction was refluxed at 50^o^C for 15 minutes followed by the addition of caesium carbonate (1 g, 3.069 x 10^-3^ mol), the reaction was left to stir overnight at 50^o^C. The resulting solution was poured into icy water (100 mL). A precipitate was formed and after vacuum filtration, the product was dried in a vacuum oven at 45^o^C overnight. The crude product (base of BNIPDPP) was recrystallized from ethanol (64.8%).

^1^H-NMR (CDCl_3_): δH 8.52– 8.50 (2H, CH aromatic protons), 8.15– 8.11 (2H, CH aromatic protons), 7.70–7.65 (2H, CH aromatic protons), 4.17– 4.14 (2, CH_2_ protons), 2.84 – 2.82 (2H, CH_2_ protons), 2.41 – 2.37 (2H, CH_2_ protons), 1.91-1.83 (2H, CH_2_ protons), 1.79-1.74 (2H, CH_2_ protons), 1.51-1.48 (2H, CH_2_ protons), 1.71-1.09 (H, CH protons) ppm.

In a round bottom flask (50 cm^3^), the free base BNIPDPP (1 g, 1.459 x 10^-3^ mol) was dissolved in DCM (20 mL) and HBr/CH_3_CO_2_H (2 mL) was added slowly. The reaction was stirred for 2 hours at room temperature to yield a precipitate. The latter was filtered off by and washed with DCM (30 mL) and ether (10 mL). The BNIPDPP salt was dried under *vacuo* at 45^o^C for 2 hours (yield 72.3%).

^1^H-NMR (CDCl_3_): δH 8.53 – 8.51 (2H, CH aromatic protons), 8.14 – 8.12 (2H, CH aromatic protons), 7.70 – 7.66 (2H, CH aromatic protons), 4.69 (2H, CH_2_ protons), 4.17 – 4.14 (2H, CH_2_ protons), 2.84 – 2.82 (2H, CH_2_ protons), 2.41 – 2.37 (2H, CH_2_ protons), 1.91-1.83 (2H, CH_2_ protons), 1.79-1.74 (2H, CH_2_ protons), 1.51-1.48 (2H, CH_2_ protons), 1.71-1.09 (H, CH proton) ppm.

HRMS (ESI): calculated for C_43_H_48_N_4_O_4_ [M+H]^+^ - 2Br: 685.3748, found: 685.3736.

**BNIPDapp, 7a.** Synthesis of N,N-dimesityl-aminopropylpiperazine. In a round bottomed flask, 1,4 bis(3-aminopropyl)piperazine (1 g, 0.0499 mol) was dissolved in 10 mL of anhydrous pyridine. The solution was stirred at 0°C (on ice) then mesitylenesulfonyl-chloride (2.19 g, 0.01003 mol, 2.01 molar excess) was added slowly, over 15 minutes. The reaction was stirred for 1 hour at 0°C (on ice) and monitored using TLC. When complete, the solution was poured into 50 mL of icy water while stirring with a glass rod and left to settle for 15 minutes to form a precipitate. The latter was filtered off, washed thoroughly with water and dried under *vacuo*, followed by recrystallisation from ethanol (36%). In a round bottomed flask, N,N-dimesityl-aminopropylpiperazine (0.3 g, 0.5357 mmol) and O-tosylpropylphthalimide (0.46 g, 1.125 mmol, 2.01 x excess) was dissolved in 6 mL of DMF. Once fully dissolved cesium carbonate (0.873 g, 2.6785 mmol) was added slowly. The solution was stirred overnight at 60°C. When complete, the solution was poured into 50 mL of icy water and stirred with a glass rod to yield a precipitate. The latter was filtered off, washed thoroughly with water and then dried under vacuum at 50°C. The crude product was recrystallized from ethanol to give BNIP-Di-Mts-aminopropylpiperazine (49% yield). In a round bottomed flask, 0.23 g of BNIP-Di-Mts-aminopropylpiperazine (0.222 mmol) was dissolved in 10 mL of anhydrous dichloromethane (DCM) followed by the addition of 1 mL of hydrobromic acid in glacial acetic acid (HBr/gCH_3_CO_2_H). The solution was left stirring for 24 hours at room temperature and monitored using TLC. When complete, the precipitate formed was filtered off and washed with 20 mL of anhydrous DCM and 5 mL of ether. The dihydro-bromic salt of bisnaphthalimidopropyl-diamino-propylpiperazine (BNIPDapp) (75% yield).

BNIPDapp: ^1^H NMR (DMSO): δ 8.5-7.5 (aromatic protons), 4.1 (CH_2_-Naphth), 2.6 (CH_2_-NH, in chain), 2.5 (CH_2_-N, from ring), 2.0 (NH) and 1.5 (CH_2_). ^13^C NMR (DMSO): δ 165.0 (C=O), 140.0–120.0 (aromatic carbons), 70.0 (CH_2_, from ring), 20.0-50.0 (CH_2_, from chain). HRMS (ESI): calculated for C_40_H_48_N_6_O_4_Br_2_, 833.1846 [M-2H-Br]^+^, found: 833.1844 [M-2H-Br]^+^

**BNIPDmPP, 12.** In a round bottomed flask, 1, 4 bis(3-aminopropyl) piperazine (0.3 g, 1.498 mmol) and 1, 8-naphthalic anhydride (3.011 mmol, 0.539 g, 2.01 molar excess) was dissolved in DMF (7 mL) followed by the addition of DBU (1 mL). The solution was stirred for 2 hours at 70°C and was monitored using TLC. When the reaction was complete, the solution was poured into 50 mL of icy water and stirred with a glass rod to form a precipitate. The precipitate was filtered off, washed thoroughly with water and dried under vacuum at 50°C for 2 hours. The product, Bisnaphthalimidopropyl-piperazine (87% yield), required no further purification for the next step. Bisnaphthalimidopropyl-piperazine (0.25 g, 0.4464 mmol) was then methylated in 25 mL of anhydrous DCM followed by the addition of methyl-iodide (2 mL). The solution was boiled under reflux overnight to yield a precipitate, which was filtered off and washed with 20 mL of anhydrous DCM. The methyl-iodide quaternary salt of bisnaphthalimidopropyl-piperazine (BNIPDmPP) (69% yield) was dried under *vacuo* at 50°C overnight.

^1^H (DMSO-d_6_): δ 8.46-8.38, 7.85-7.79 (aromatic protons), 4.08 (triplet, CH_2_), 4.00 (triplet, CH_2_), 3.46-3.41, 3.34-3.26 (multiplet, 2 x CH_2_), 2.93 (singlet, 3H, CH_3_), 2.76-2.72 (2H, CH_2_), 2.57-2.54 (2H, CH_2_), 2.09-2.05 (multiplet, 2H, CH_2_), 1.74-1.71 (multiplet, 2H, CH_2_).

HRMS (ESI): calculated for C_35_H_34_N_4_O_4_ :575.2658, found: 575.2653 [M+H]^+^.

**BCNIPP, 13.** In a round bottomed flask, (0.596 g, 3.01 mmol) of 4-chloro-1,8-naphthalic anhydride was dissolved in 7 mL of DMF. 0.3g (1.498mmol) of bis(3-aminopropyl)piperazine were then added to the solution, followed by 0.7 mL of DBU. The solution was left to stir at 70ºC for 48 hours. When complete, the reaction solution was poured in cold water (100 mL) while stirring to form a precipitate as the product. This precipitate was then filtered with a Buchner funnel and washed up with water. The solid obtained was dried under *vacuo*, to yield BCNIPP (68% yield).

^1^H NMR (CDCl_3_): δ = 8.56-8.40; 7.76-7.73 (naphthalimido ring protons), 4.12 (triplet, CH_2_-naphthalimide), 2.21 (triplet, CH_2_ piperazine ring and CH_2_-N), 1.77 (pentet, CH_2_). ^13^C NMR (CDCl_3_): δ = 163.5 (4-chloro-naphthalimide carbonyl), 138.97-121.75 (4-chloro-naphthalimide carbons), 56.07 (CH_2_-naphthalimide), 53.01 (piperazine CH_2_), 39.06 (CH_2_-piperazine), 24.89 (CH_2_). HRMS (ESI): calculated for C_34_H_30_Cl_2_N_4_O_4_: 629.1644 (M+H); found: 629.1716.

**Screening of enzyme inhibitors**

A variety of the bisnaphthalimidopropyl (BNIPs) derivatives, as well as the newly synthetized derivatives were evaluated as potential TcSir2rp1 inhibitors. The enzymatic reactions were performed using a commercially available CycLex SIRT1/Sir2 deacetylase fluorimetric kit (CycLex Co. Ltd., Nagano, Japan) in the absence and presence of the various inhibitors, 200 µM NAD^+^ and 10 µM of peptide substrate as previously described [46]. The inhibition is expressed in percentage and was calculated as the ratio of velocity for the linear portion of the reaction, normalized with a no drug control and a reference drug control (nicotinamide at 2 mM).

**Supporting Information Legends**

**S1 Table. Enzymatic inhibition of hSIRT1 by newly synthetized BNIPs.**

| Compound | hSIRT1 Inhibition at 10 µM  (% ± SD) |
| --- | --- |
| 12 | N.I. |
| 13 | 15 ± 4 |
| 1a | 1 ± 1 |
| 1b | 3 ± 4 |
| 1c | N.I. |
| 6a | 3 ± 4 |
| 6b | 44 ± 8 |
| 6c | 8 ± 1 |
| 7a | N.I. |
| 9a | 3 ± 4 |

*N.I. = no inhibition. Values are means ± SD n=2
