# Supplementary figures and images for: Inhibitors of Trypanosoma cruzi Sir2 related protein 1 as potential drugs against Chagas disease
Source: PLoS Negl Trop Dis. 2018 Jan 22;12(1):e0006180. doi: 10.1371/journal.pntd.0006180 (PMC5794198; doi:10.1371/journal.pntd.0006180)

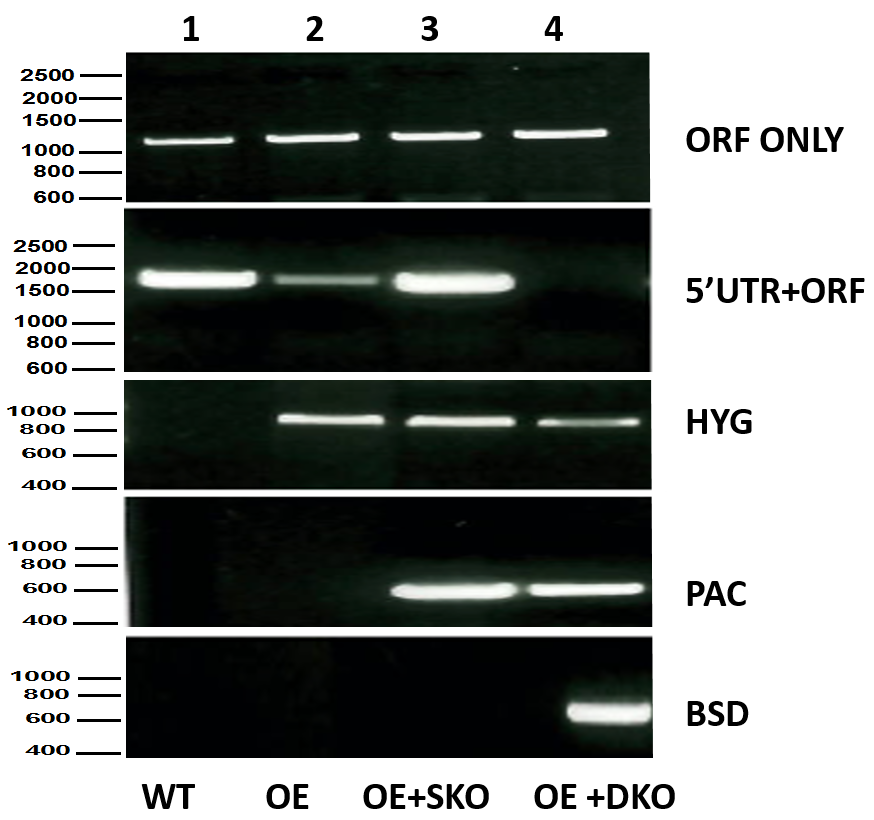

Supplement: S2 Fig — PCR validation of the genetically manipulated cell-lines: Lane 1: WT; Lane 2: over-expressing cell-line pTcINDEX TcSir2rp1: Hyg; Lane 3: over-expressing and single knockout cell-line TcSir2rp1ΔTcSir2rp::Pac pTcINDEX TcSir2rp1: Hyg; Lane 4: over-expressing and double knockout cell-line TcSir2rp1ΔTcSir2rp::Pac, Bsd pTcINDEX TcSir2rp1: Hyg. Primers used for ORF only are the TcSir2rp1 ORF forward and reverse primers, expected PCR product 1092bp. Primers used for the 5’UTR+ORF are the 5’ UTR F1 and TcSir2rp1 reverse ORF, expected size 1683bp. Forward and reverse primers used for hygromycin resistance cassette (HYG), expected size 1041bp. Forward and reverse primers used for puromycin resistance cassette (PAC), expected size 610bp. Forward and reverse primers used for blasticidin resistance cassette (BSD), expected size 432bp. Forward and reverse ORF primers (5’-TATGCGGCCGCATGAATCAAGATAACGCCAAC-3’ and 5’- TATGGATCCTTATTTTCGGTCTGTCTGTGTGTACATG-3’ respectively. 5’ and 3’ UTR Primers respectively (F1: 5’-TATGCGGCCGCAGGAACCCACCACTTC-3’; R1: 5’- CGTTTAAACTTACGGACCGTCAAGCTTTGGGAAGAAGTAATCCACCT-3’; F2: 5’-GACGGTCCGTAAGTTTAAACGGGATCCACCGAAAATAAGAGGA-3’; R2: 5’-TATGCGGCCGCGATGCTCTTCATATTTATCTTGC-3’. (TIF) [file pntd.0006180.s003.tif]

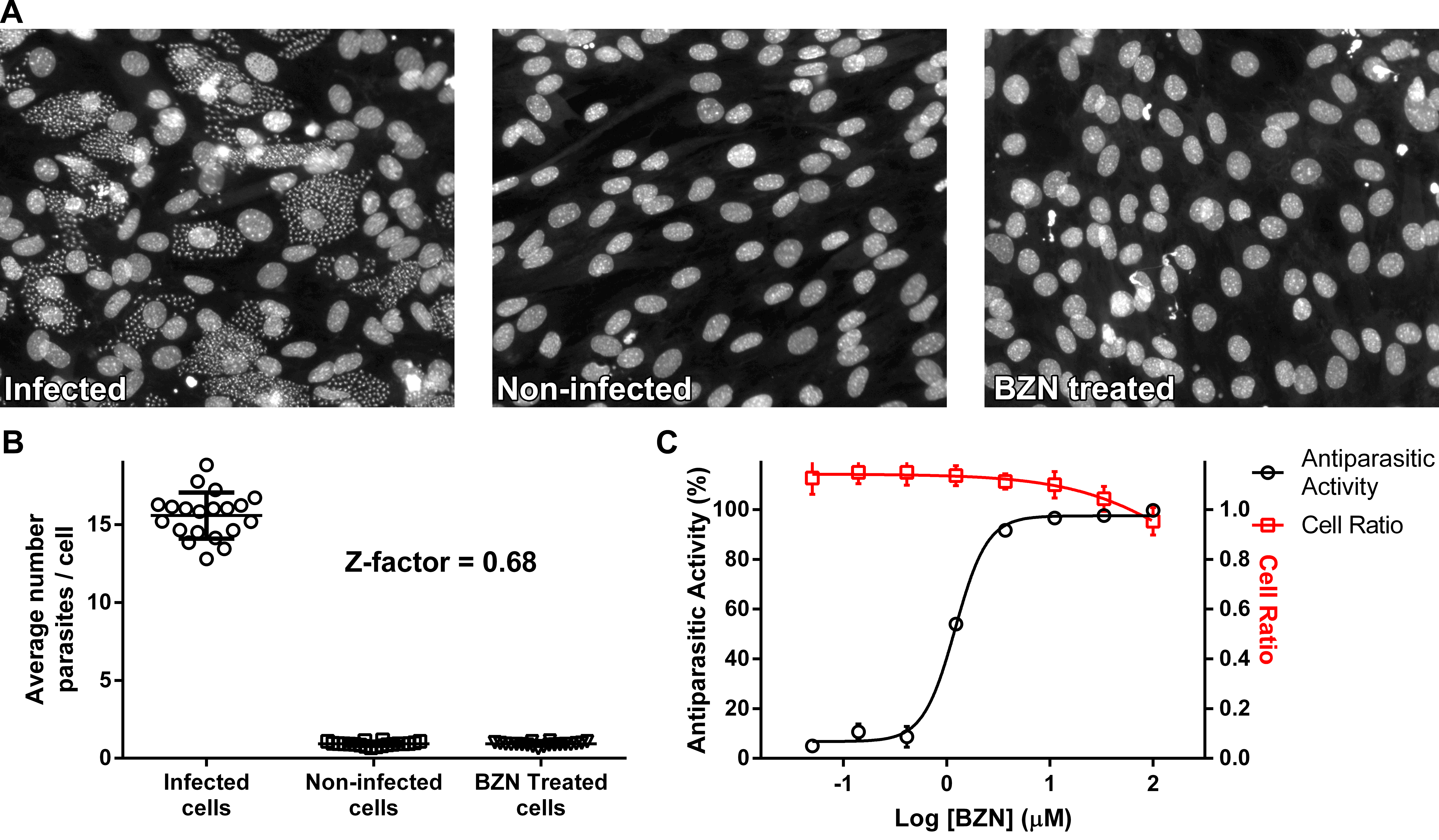

Supplement: S3 Fig — A) Representative images of the control conditions used in the assay: infected cells, non-infected cells and infected cells treated with 100 μM of benznidazole (BZN). B) Statistical validation of the screening assay based on the readout of parasites per cell, with a Z value of 0.68. C) Dose-response curve against the reference drug benznidazole, demonstrating and EC50 in line with previously published values [62]. (TIF) [file pntd.0006180.s004.tif]

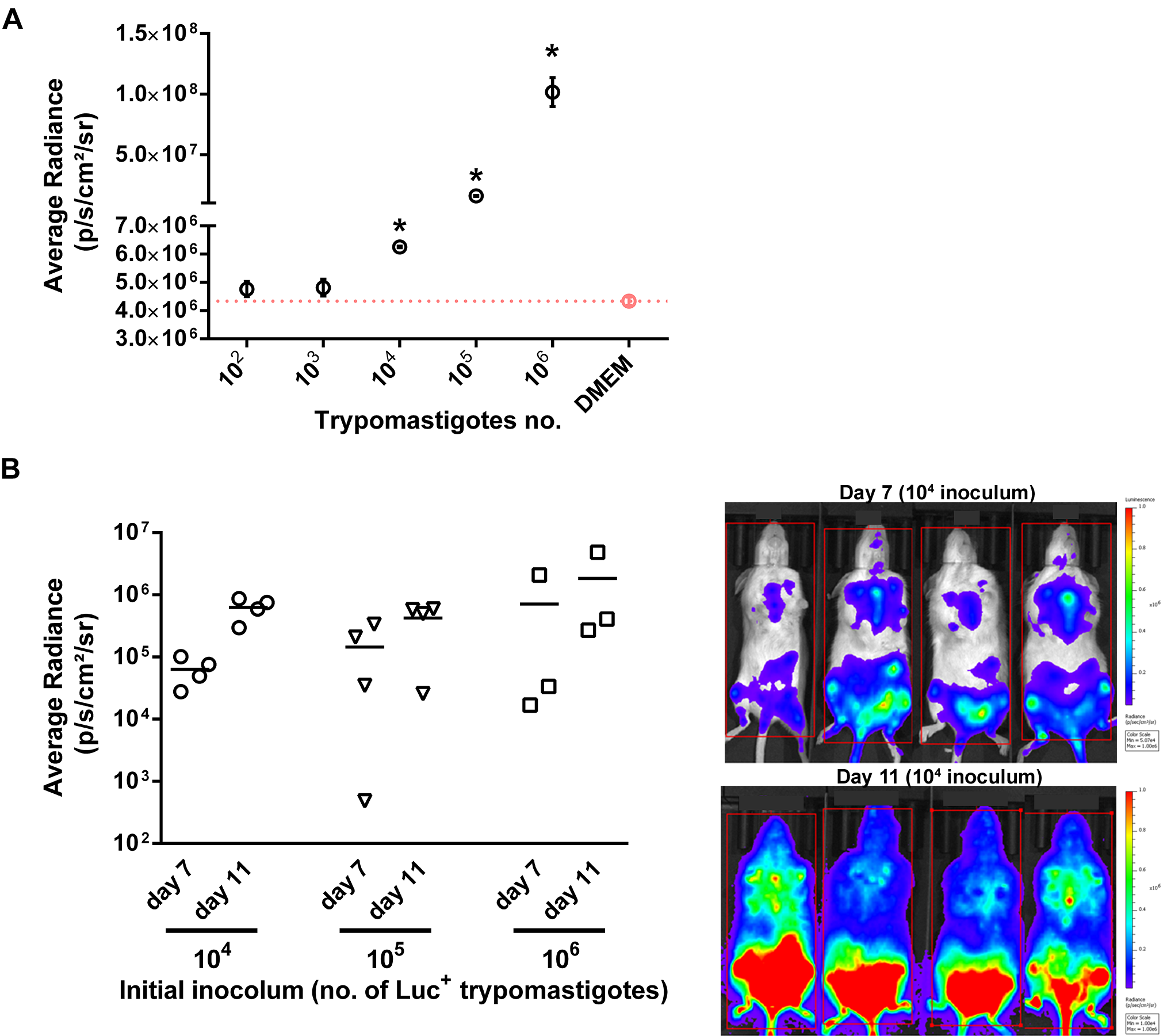

Supplement: S4 Fig — A) In vitro detection limit of Luc+ trypomastigotes in a 96-well plate incubated with luciferin and imaged using the IVIS LUMINA LT. Circles and vertical lines represent the average ± standard deviation of the average radiance of quadruplicates. Statistical analysis was performed by standard t-test relative to luciferin background with no parasites. Significance is shown in asterisks (*, p-value ≤ 0.05). B) Comparison of different inocula of Luc+ trypomastigotes (104, 105, 106) tested in BALB/c mice infections by quantification of the signal over 5 minutes (average radiance, photons/sec/cm2/sr) C) Representative images of the anesthetized bio-imaged mice infected with 104 trypomastigotes at the time points before and after treatment (day 7 and 11 after infection, respectively). (TIF) [file pntd.0006180.s005.tif]

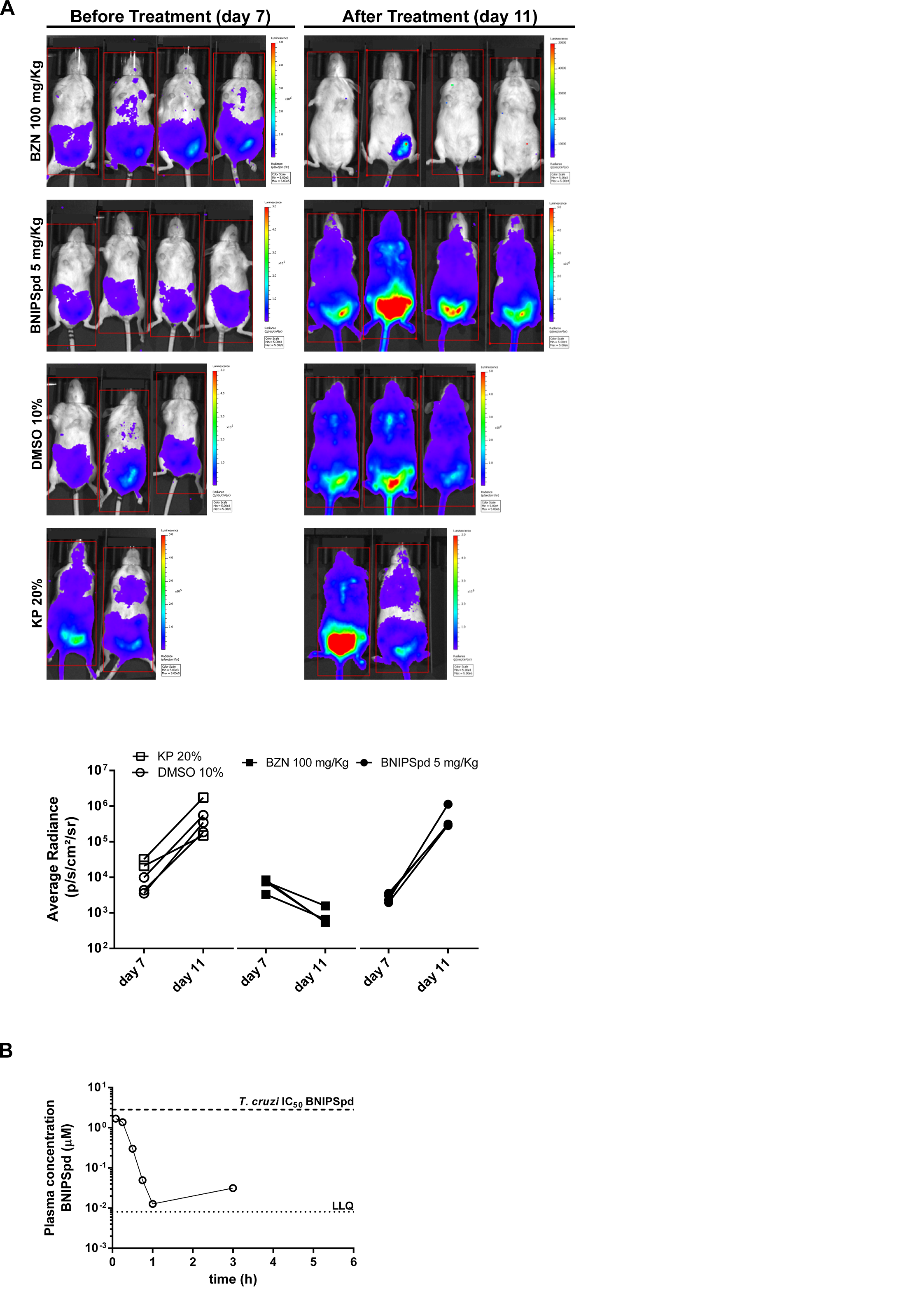

Supplement: S5 Fig — A) Mice were infected with 104 Luc+ trypomastigotes by intraperitoneal injection. Treatments (BZN, benznidazole at 100 mg/kg/day per os, and BNIPSpd (9) at 5 mg/kg/day by intravenous injection) were initiated 7 days after infection, as well as the respective controls (KP, Kolliphor HS 15 20% per os and DMSO 10% by intravenous injection). Imaging was performed before treatment, at 7 days post-infection and after treatment, at 11 days post-infection, using an IVIS LUMINA LT and upon injection of 2.1 mg luciferin. In the lower panel, bioluminescence signal of whole mice represented in average radiance (photons/sec/cm2/sr) quantified before and after treatment. Data representative of two independent experiments. B) Snapshot Pharmacokinetics of BNIPSpd (9) in BALB/c mice by quantification of BNIPSpd (9) in the blood of mice by UHPLC-MS/MS ESI+ at different time-points after administration of a 5 mg/kg dose by intravenous injection. Data is the average of two independent experiments. The dashed line represents the value of EC50 for BNIPSpd (9) in the in vitro assay against T. cruzi amastigotes, and the dotted line represents the lower limit of quantification of the technique. (TIF) [file pntd.0006180.s006.tif]

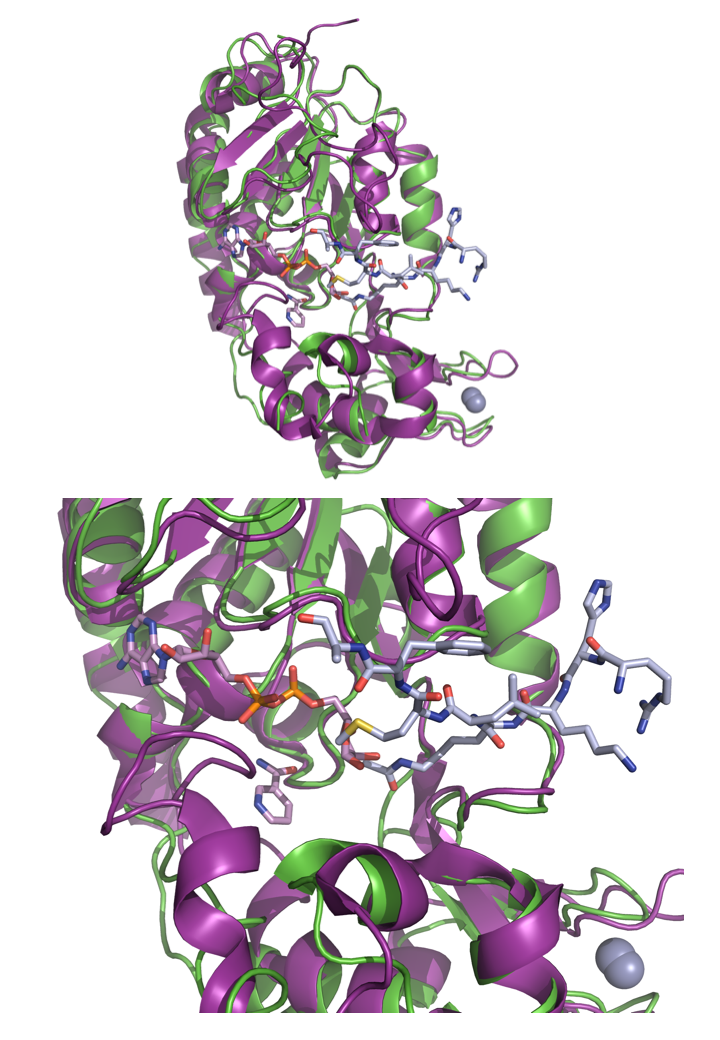

Supplement: S6 Fig — TcSir2rp1 structure in green, with p53 peptide carbons in pale blue. Human SIRT2 (4rmj) structure in purple, with carbons of ligands ADP and nicotinamide in pale pink. (TIFF) [file pntd.0006180.s007.tiff]

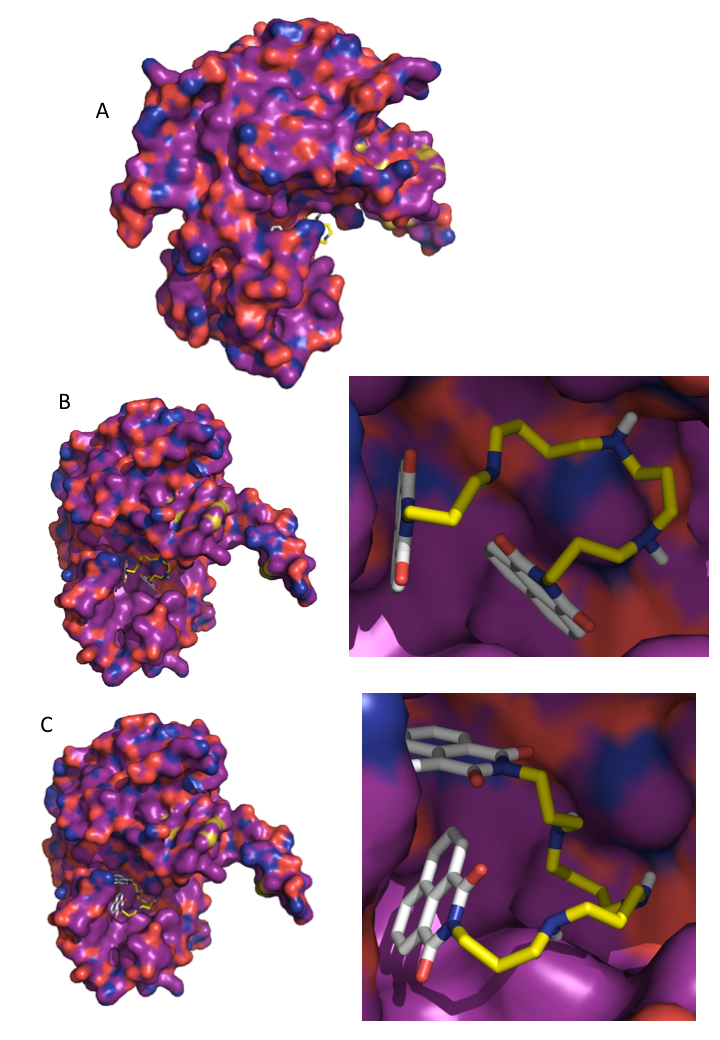

Supplement: S7 Fig — A) image shows the same orientation as TcSir2rp1 with compound 9 (Fig 6B and 6C), B) is the same model rotated by 90° to view putative ligand binding pocket, with a zoomed in image on compound 9. C) images showing an alternative mode of compound 9 binding.All of these have similar binding affinities to those observed for TcSir2rp1. (TIFF) [file pntd.0006180.s008.tiff]

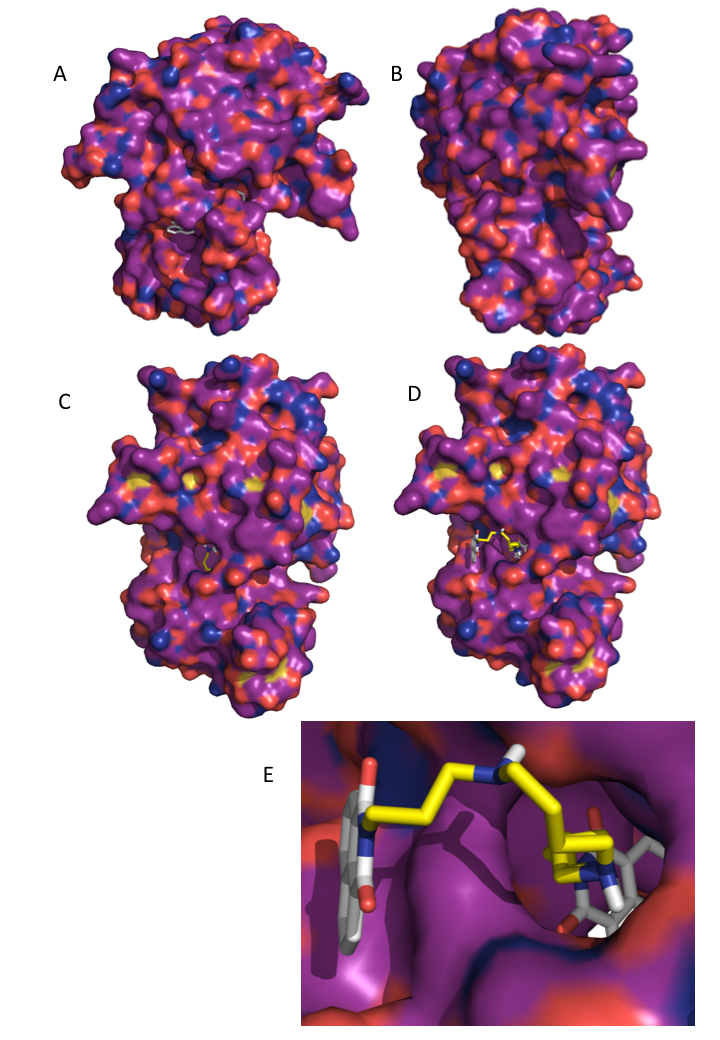

Supplement: S8 Fig — A) image shows the same orientation of TcSir2rp1 with compound 9 (Fig 6B and 6C), B) is same model rotated 90°. C) is the same model turned by an additional 90°. D) is the same rotation as C, but with an alternative binding mode by compound 9, which is zoomed in on compound 9 (E). All of these have similar binding affinities to those observed for TcSir2rp1. (TIFF) [file pntd.0006180.s009.tiff]

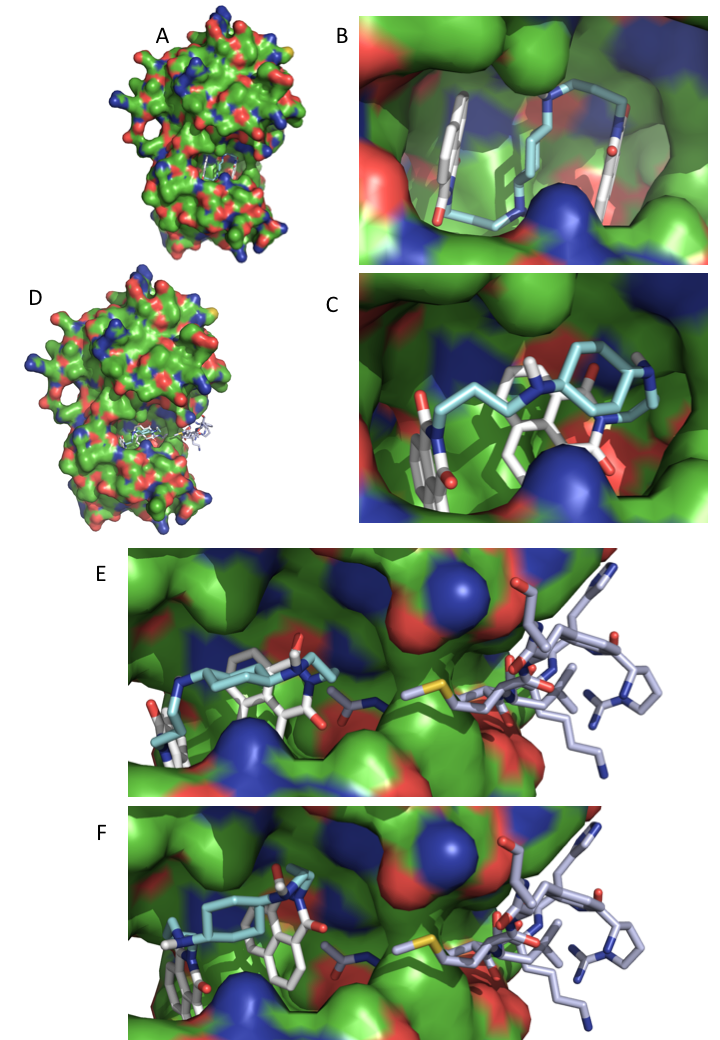

Supplement: S9 Fig — A) image shows the same orientation of TcSir2rp1 with compound 9 (Fig 6B and 6C), but with 1a docked, which is zoomed in on (B), and with an alternative binding orientation (C). D) Shows TcSir2rp1 in the presence of p53 and docked compound 1a, which is zoomed in on (E), and with an alternative binding orientation (F). These all have very similar binding affinities 8–11 kcal/mol. (TIFF) [file pntd.0006180.s010.tiff]

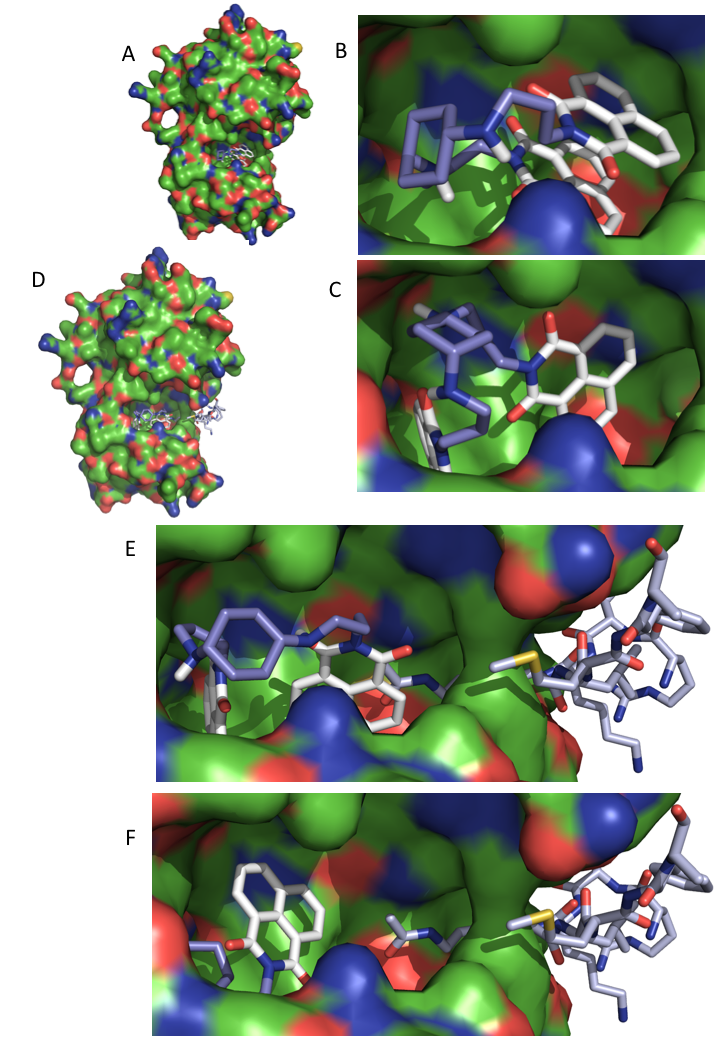

Supplement: S10 Fig — A) image shows the same orientation of TcSir2rp1 with compound 9 (Fig 6B and 6C), but with 1b docked, which is zoomed in on (B), and with an alternative binding orientation (C). D) Shows TcSir2rp1 in the presence of p53 and docked compound 1a, which is zoomed in on (E), and with an alternative binding orientation (F). These all have very similar binding affinities 8–11 kcal/mol. (TIFF) [file pntd.0006180.s011.tiff]
